# Synthesis of tricyclic fused pyrrolidine nitroxides from 2-alkynylpyrrolidine-1-oxyls

**DOI:** 10.3762/bjoc.22.22

**Published:** 2026-02-19

**Authors:** Mark M Gulman, Yuliya F Polienko, Sofia Yu Trakhininа, Yuri V Gatilov, Tatyana V Rybalova, Sergey A Dobrynin, Igor A Kirilyuk

**Affiliations:** 1 N. N. Vorozhtsov Novosibirsk Institute of Organic Chemistry SB RAS, Lavrentiev Ave. 9, Novosibirsk, 630090, Russia; 2 Novosibirsk State University, Pirogova Str. 2, Novosibirsk, 630090, Russia

**Keywords:** annulated tricyclic system, nitroxide, pyrazole, pyrrolidine, triazole

## Abstract

Rotational correlation time is a key parameter for organic radical contrast agents (ORCA) for magnetic resonance imaging (MRI). Design of polycyclic systems with incorporated nitroxide moieties in which rotation of the radical separately from the framework is impossible is one of the ways to improve properties of ORCA. Feasibility of the synthesis of rigid 3b,4,5,6,6a,7-hexahydropyrrolo[2',3':3,4]pyrrolo[1,2-*c*][1,2,3]triazole and 3b,4,5,6,6a,7-hexahydropyrrolo[2',3':3,4]pyrrolo[1,2-*b*]pyrazole ring systems with incorporated nitroxide moiety from 2-alkynyl-substituted pyrrolidine nitroxides was studied. These nitroxides have been prepared via intramolecular Huisgen cycloaddition or intramolecular alkylation in 2-pyrazolyl derivatives prepared by Michael addition–cyclocondensation of the corresponding alkynones with hydrazine. The reduction kinetics by ascorbate showed that the formation of the rigid tricyclic framework does not lead to a significant increase in stability of the radical center to chemical reduction.

## Introduction

Stable nitroxides are functional components of many high-tech materials, such as energy storage and organoelectronics devices [1–8], catalysts [9–10], bioactive coatings and nanoparticles [11–12], organic radical contrast agents (ORCAs) for magnetic resonance imaging (MRI) [13–14], etc. For these applications, numerous nitroxides are incorporated into macromolecular or nanosized supramolecular structures, which modulate nitroxide properties. For example, the efficiency of ORCA (relaxivity) directly depends on the rotational correlation time of the radicals attached to the scaffold [13–15]. Large structures in which rotation of the radical separately from the framework is impossible could be particularly promising. Rigid polycyclic fused systems with incorporated nitroxide moieties could be one of the possible ways to achieve the above feature.

We recently found that the reaction of sterically hindered 3-hydroxymethyl-2-ethynylpyrrolidine-1-oxyls with nucleophilic agents can lead to the formation of condensed systems involving the substituent at position 3 of the pyrrolidine ring [16]. Alkynes are broadly used in the synthesis of various heterocyclic compounds, and participation of neighboring functional groups often leads to formation of complex polycyclic systems [17–19]. In this study, we aimed to construct rigid tricyclic condensed systems with an integrated nitroxyl radical fragment from 3-substituted 2-ethynylpyrrolidine-1-oxyls. The desired tricyclic nitroxides were prepared via intramolecular Huisgen cycloaddition or via one-pot Michael addition–cyclocondensation reaction with hydrazine with subsequent intramolecular alkylation of the resulting pyrazoles.

## Results and Discussion

### Synthesis

**Scheme 1:**
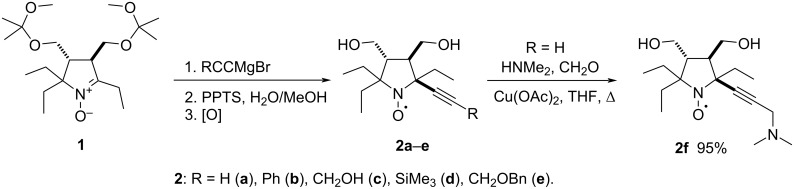
Synthesis of nitroxides **2a**–**f**.

We have earlier reported on the synthesis of 2-alkynylpyrrolidine-1-oxyls **2a**–**c** via addition of the corresponding alkynylmagnesium bromides to nitrone **1** [20]. Radicals **2d** and **2e** were prepared in analogy to the known procedure using trimethylsilylacetylene and benzyl propargyl ether as the terminal alkynes (Scheme 1). Nitrone **1** was treated with a 10-fold excess of alkynylmagnesium bromide prepared in situ via metalation of trimethylsilylacetylene or benzyl propargyl ether with ethylmagnesium bromide. After quenching, removal of the MOP protecting group, and oxidation by atmospheric oxygen the nitroxides **2d** and **2e** were isolated in 64% and 66% yields, respectively.

Terminal alkynes can be converted into propargylamines via A^3^-coupling reaction [21]. In analogy to a literature procedure [22], heating of radical **2a** in a mixture of dimethylamine, formalin, and tetrahydrofuran in the presence of copper(II) acetate afforded the corresponding dimethylamino derivative **2f**.

**Figure 1:**
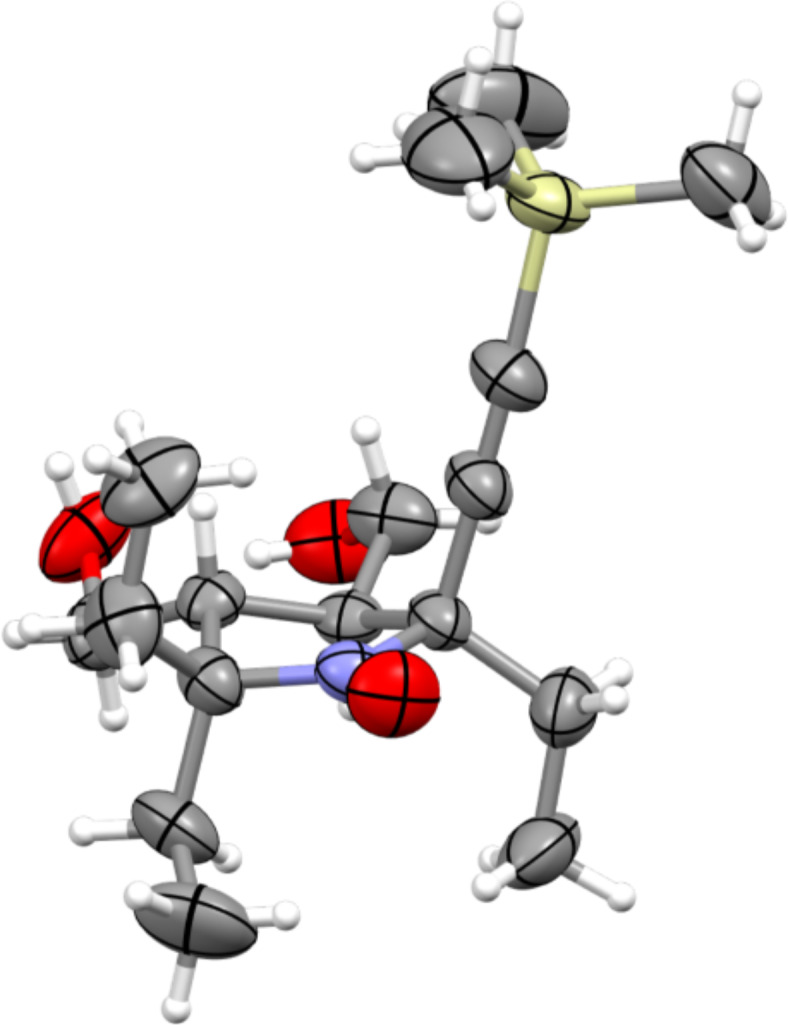
X-ray structure of nitroxide **2d**.

To confirm the structure of the novel nitroxides **2d**–**f**, the samples of the radicals were reduced to the corresponding diamagnetic amines using a Zn/CF_3_COOH system in CD_3_OD at 63 °C, according to a literature protocol [23] and the ^1^H NMR spectra were recorded. The spectra showed similarity to those of previously described for radicals **2a**–**c** [20] with characteristic signals of (2*R*)-2,5,5-triethyl-3,4-bis(hydroxymethyl)pyrrolidines. An additional singlet at 0.21 ppm in the spectrum of **2d** was assigned to the hydrogen atoms of the trimethylsilyl group. The spectrum of compound **2e** displayed two singlets at 4.33 ppm and 4.62 ppm (2H each), assigned to the methylene groups of the propargyl and benzyl fragments, respectively, and a multiplet of phenyl group in the range of 7.30–7.40 ppm (5H). Successful aminomethylation of the ethynyl group was confirmed by the appearance of two singlets at 3.01 ppm (6H) and 4.28 ppm (2H), attributed to the protons of the dimethylamino group and the methylene protons of the propargyl moiety. The structure of nitroxide **2d** was confirmed by single crystal X-ray analysis. (Figure 1, CCDC 2512649).

**Scheme 2:**
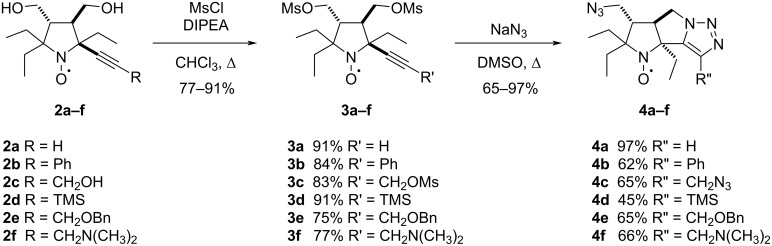
Synthesis of mesyl **3a**–**f** and triazole **4a**–**f** derivatives.

The nitroxides **2a**–**f** were used to synthesize tricyclic nitroxides **4a**–**f**. The mesylation was carried out in the presence of DIPEA in chloroform under reflux (Scheme 2). These conditions ensured complete conversion in 30 minutes.

The IR spectra of nitroxides **3a**–**f** exhibit intense absorption bands in the ranges of 1354–1358 cm^−1^ and 1174–1178 cm^−1^, corresponding to the asymmetric and symmetric vibrations of the sulfonate group, respectively [24]. The ^1^H NMR spectra of **3a**,**b**,**d**–**f** (Zn/CF_3_COOH system in CD_3_OD) showed appearance of a singlet of methanesulfonate hydrogens in the region from 2.78 to 3.17 ppm. The NMR spectra were not recorded for **3c** because of heavy resinification upon the sample preparation.

The literature data on reactivity of 5-azidopentyne derivatives in intramolecular Huisgen cycloaddition reactions are contradictory. Some authors successfully obtained 5-azidopentyne derivatives upon nucleophilic substitution at 80 °C in DMF, and additional heating at 170 °C was necessary for cyclization to triazoles to occur [25]. However, there are also examples where the corresponding triazoles were isolated instead of 5-azidopentyne derivatives under the same conditions (DMF, 80 °C) [26].

**Figure 2:**
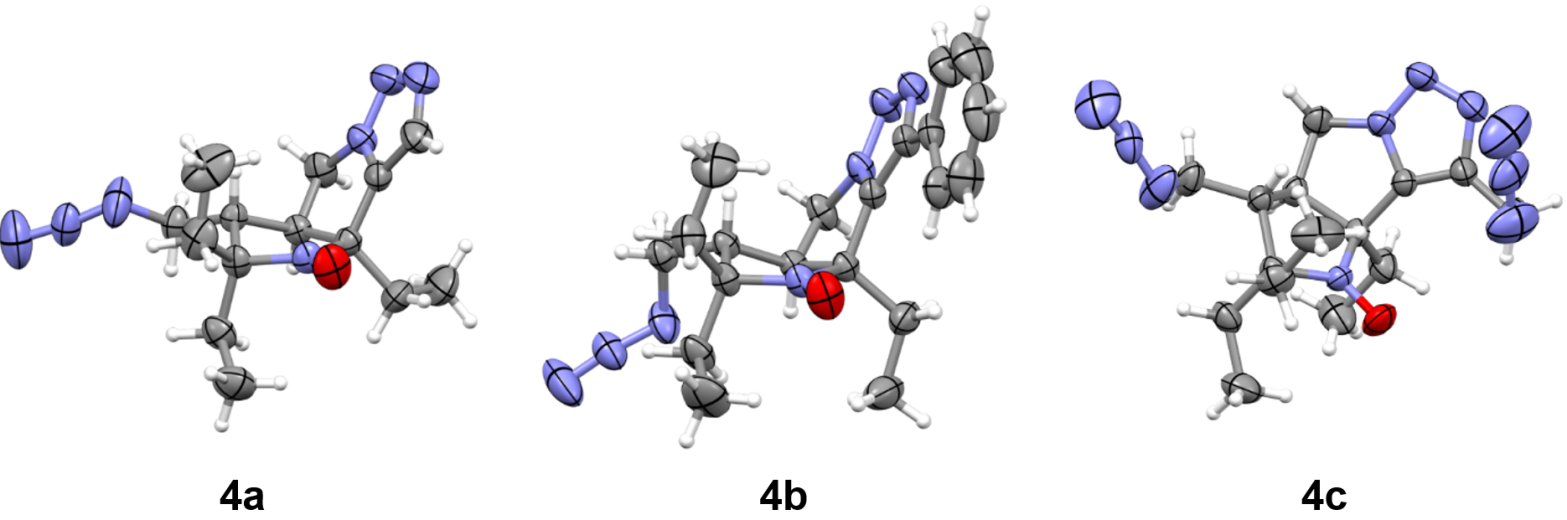
X-ray structures of nitroxides **4a**–**c**.

The nitroxides **3a**–**f** were treated with excess of NaN_3_ in milder conditions, in DMSO at 60 °C, and a single product was isolated from the reaction mixtures in each case. The IR spectra of the isolated compounds **4a**–**f** showed characteristic absorption bands to the azido group vibrations at 2100–2116 cm^−1^. Absorption bands in the ranges of 1410–1420 cm^−1^ and 1170–1190 cm^−1^ were also observed, which can be assigned to the out-of-plane bending (wagging, ω) and breathing vibrations of the triazole ring, respectively [27]. The X-band EPR spectra of nitroxides **4a**–**f** revealed remarkable difference with those of **3a**–**f** (see Table 1). In analogy to previously reported parameters for **2a**–**c** [20], EPR spectra of trans-3,4-disubstituted 2,2,5-triethyl-5-ethynylpyrrolidine-1-oxyls **2d**–**f** and **3a**–**f** correspond to triplet of doublets pattern with hfc *a*_N_ = 1.53–1.58 mT at the nitrogen atom of nitroxide group and an additional hfc *a*_H_ = 0.21–0.23 mT at one of the methylene hydrogens of the ethyl groups [20,28]. The spectra of tricyclic nitroxides **4a**–**f** are characterized with smaller triplet splitting, *a*_N_ = 1.43–1.46 mT, while hfc on hydrogen reach 0.26–0.31 mT, with the exception of **4b** (0.22 mT). The structures of nitroxides **4a**, **4b**, and **4c** were confirmed by single crystal X-ray crystallographic analysis (Figure 2, CCDC 2512650–2512652).

**Table 1:** Reduction rate constants *k*_2_ and EPR spectral parameters of nitroxides **2a**–**f, 4a**–**f, 9c**.

Nitroxide	*k*_2_∙10^1^, M^−1^s^−1^	*a*_N_, mT (±0.005)	*a*_H_, mT (±0.005)	Line width, mT (±0.001)

**2a**	2.38 ± 0.01	1.53	0.23	0.006
**2b**	1.86 ± 0.05	1.53	0.21	0.006
**2c**	1.35 ± 0.05	1.54	0.21	0.006
**2d**	2.50 ± 0.05	1.53	0.21	0.006
**2e**	1.46 ± 0.06	1.53	0.21	0.006
**2f**	2.11 ± 0.05	1.53	0.23	0.006
**4a**	3.13 ± 0.02	1.47	0.26	0.007
**4b**	1.71 ± 0.06	1.45	0.22	0.007
**4c**	3.53 ± 0.05	1.45	0.26	0.007
**4d**	4.39 ± 0.10	1.45	0.27	0.007
**4e**	0.81 ± 0.01	1.43	0.26	0.007
**4f**	1.32 ± 0.01	1.46	0.31	0.006
**9с**	0.85 ± 0.01	1.49	0.25	0.007

**Scheme 3:**
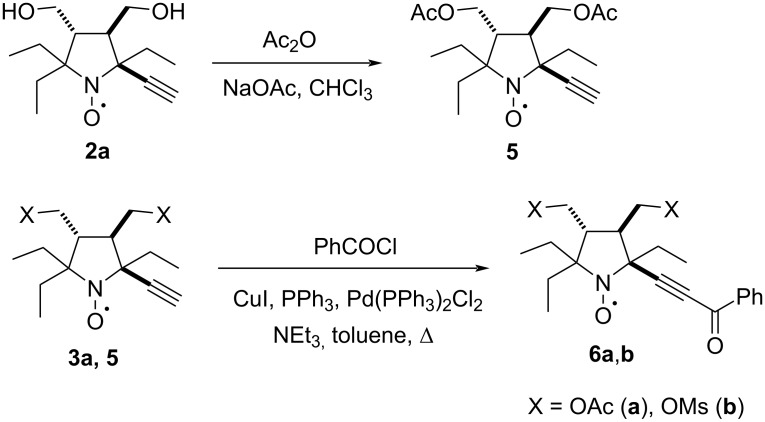
Synthesis of alkynones **6a**,**b**.

Similar rigid condensed systems can be constructed on the basis of pyrazole ring. The reaction of conjugated alkynones with hydrazine is a common pathway to pyrazoles [29]. Alkynones, in turn, can be prepared by Sonogashira acylation of terminal alkynes [30]. To prevent formation of mixtures due to incomplete benzoylation of hydroxymethyl groups under the conditions of Sonogashira cross-coupling, nitroxide **2a** was treated with acetic anhydride in the presence of sodium acetate, yielding the diacyl derivative **5**. Radicals **5** and **3a** were heated with benzoyl chloride and triethylamine in toluene in the presence of a catalytic system comprising PPh_3_, CuI, and Pd(PPh_3_)_2_Cl_2_. This procedure afforded alkynones **6a**,**b** in the yields of 75% and 44%, respectively (Scheme 3).

In the IR spectra of **6a**,**b** intense bands were observed at 2212–2214 and 1645–1647 cm^−1^, assigned to vibrations of the triple bond, and the conjugated carbonyl group, respectively. The elemental analyses data and high-resolution mass spectra (HRMS) of **6a,b** were in agreement with the assigned structure.

**Scheme 4:**
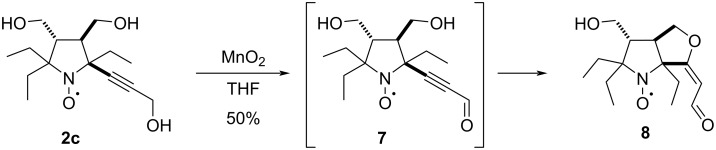
Synthesis of vinyl ether **8**.

**Figure 3:**
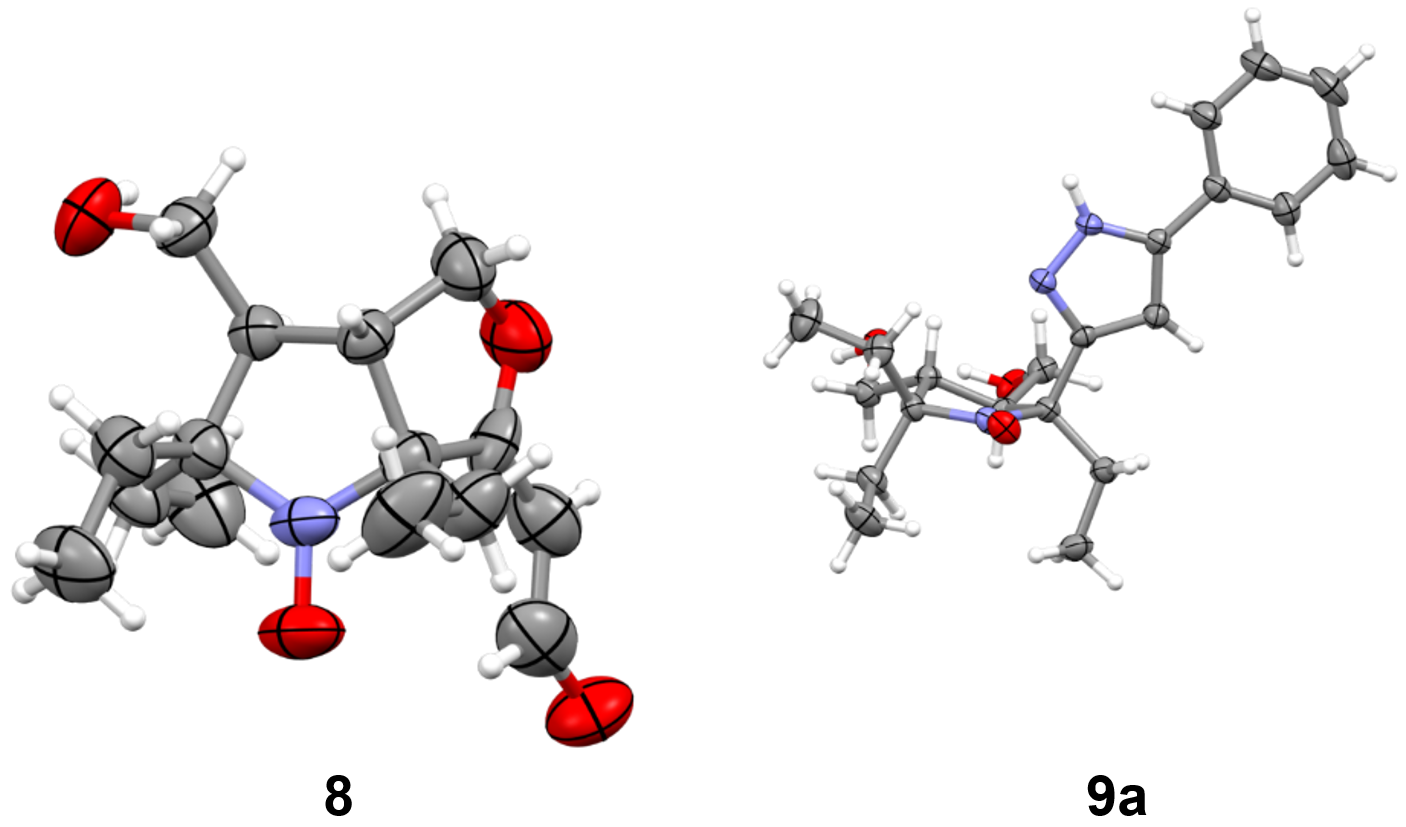
X-ray structures of nitroxides **8** and **9a**.

Oxidation of propargyl alcohols is another way to α,β-acetylenic carbonyl compounds [31]. Mild oxidation of propargyl alcohol **2c** with activated manganese dioxide in tetrahydrofuran gave vinyl ether **8**, which was isolated with 50% yield (Scheme 4). The structure of the product **8** was confirmed by X-ray crystallographic analysis (Figure 3, CCDC 2512653). Formation of **8** apparently occurs via cyclization of alkynal **7**. The formation of vinyl ethers has been previously described for radicals **2a** and **2b**, but these cyclizations required heating in the presence of a base [16].

**Scheme 5:**
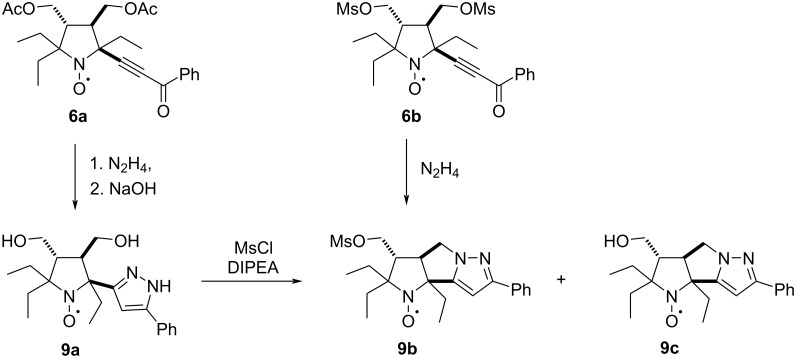
Synthesis of pyrazole derivatives **9a**–**c**.

To obtain pyrazole derivatives, alkynones **6a**,**b** were treated with hydrazine (Scheme 5). Formation of the pyrazole ring in the reaction of **6b** was accompanied by partial hydrazinolysis of the acetoxy groups and reduction of the nitroxyl fragment to the corresponding hydroxylamine. Primary products were stirred with a solution of NaOH in methanol under aerobic conditions to achieve complete hydrolysis and regeneration of the nitroxide group. Pyrazole **9a** was isolated with an overall yield of 33%, and its structure was confirmed by single crystal X-ray crystallographic analysis (Figure 3, CCDC 2512654).

Treatment of **6b** with hydrazine resulted in the formation of a condensed tricyclic ring system and nitroxide **9b** was isolated in 42% yield. This nitroxide was alternatively prepared via mesylation of **9a**. The yield of **9b** in this reaction depends on excess of the reagent. Heating of **9a** with five equivalents of MsCl and excess of DIPEA afforded **9b** in 65% yield. When a 1.5-fold excess of mesyl chloride per hydroxy group was used, another nitroxide **9c** was isolated in 26% yield along with **9b** (43% yield).

**Figure 4:**
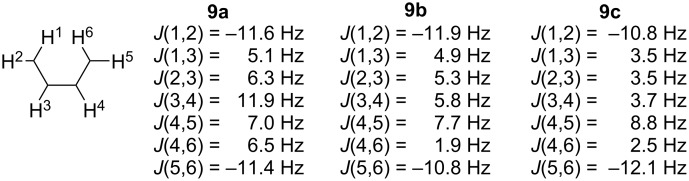
Spin–spin coupling constants for reduced nitroxides **9a**–**c**.

The IR spectra of the pyrazole derivatives **9a**–**c** exhibit absorptions at 688–699 cm^−1^ and 763–770 cm^−1^, assigned to out-of-plane bending modes of the pyrazole ring [32]. The ^1^H NMR spectra of the reduction products of pyrazoles **9a**–**c** were recorded after reduction using the Zn/CF_3_COOH system in CD_3_OD. The full spectral line shapes were simulated using the gNMR program to assign the signals and to obtain the coupling constants [33]. Direct comparison of the spin–spin coupling constants within the six-spin systems of the non-annulated nitroxide **9a** with its annulated counterparts **9b**,**c** revealed reduced dynamic averaging for one of the two methylene groups in **9b**,**c**. The similar values of constants *J*(1,3), *J*(2,3), *J*(4,5), and *J*(4,6) in **9a** reflect rapid, nearly degenerate rotation of its exocyclic methylene groups. In contrast, the disparate values of *J*(4,5) and *J*(4,6) in nitroxides **9b**,**c** demonstrate that this methylene fragment has a more fixed conformation, consistent with the annulated structure (Figure 4).

Nitroxides are known to decay in biological systems; the major mechanism is chemical reduction with cellular antioxidants (ascorbic acid and glutathione) and enzymatic systems [34]. The rate of reduction is an important factor for application of a nitroxide as an EPR spin probe or as a component of a MRI contrast agent. The bimolecular rate constants of reduction of radicals **2a**–**f**, **4a**–**f** and **9c** with ascorbate were measured with addition of the glutathione system to suppress reverse reaction [35]. The results are given in Table 1. Alkynyl and heteroaromatic derivatives showed no significant differences in the reduction kinetics with the values of the reduction rate constants (0.08–0.44 M^−1^s^−1^) comparable in scale to those reported for tetramethyl nitroxides of the pyrrolidine and pyrroline series (0.1–0.3 M^−1^s^−1^). These rates are more than two orders of magnitude higher than those of tetraethyl nitroxides of the pyrrolidine series (0.001–0.0001 M^−1^s^−1^) [28–29]. This could result from the electron-withdrawing effect of alkyne or heteroaromatic substituent. Moreover, it can be noted that rigid tricyclic systems apparently do not have a screening effect on the radical center.

## Conclusion

In this work we showed that formation of rigid tricyclic annulated systems from bifunctional nitroxides bearing alkynyl and another functional group in neighboring positions is feasible. The new tricyclic nitroxyl radicals of the pyrrolidine series annulated with 5,6-dihydro-4*H*-pyrrolo[1,2-*c*][1,2,3]triazole or 5,6-dihydro-4*H*-pyrrolo[1,2-*b*]pyrazole systems have been synthesized and characterized. Despite these nitroxides showed relatively low stability to reduction, the general strategy suggested here can be applied for the synthesis of various polycyclic systems with incorporated nitroxide.

## Supporting Information

File 1 Experimental protocols, copies of the ^1^H NMR, IR and EPR spectra and X-ray analysis data.

File 2 Crystallographic information of selected compounds.

## Data Availability

All data that supports the findings of this study is available in the published article and/or the supporting information of this article.
