# Supplementary material for: Synthesis of tricyclic fused pyrrolidine nitroxides from 2-alkynylpyrrolidine-1-oxyls
Source: Beilstein J Org Chem. 2026 Feb 19;22:344–51. doi: 10.3762/bjoc.22.22 (PMC12927486; doi:10.3762/bjoc.22.22)
Supplement: File 1 — Experimental protocols, copies of the 1H NMR, IR and EPR spectra and X-ray analysis data. [file Beilstein_J_Org_Chem-22-344-s001.pdf]

BEILSTEIN JOURNAL OF ORGANIC CHEMISTRY

Supporting Information

for

Synthesis of tricyclic fused pyrrolidine nitroxides from 2-alkynylpyrrolidine-1-oxyls

Mark M. Gulman, Yuliya F. Polienko, Sofia Yu. Trakhinina, Yuri V. Gatilov, Tatyana V. Rybalova, Sergey A. Dobrynin and Igor A. Kirilyuk

Beilstein J. Org. Chem. **2026,** *22,* 344–351. doi:10.3762/bjoc.22.22

Experimental protocols, copies of the ^{1}H NMR, IR and EPR spectra and X-ray analysis data

License and Terms: This is a supporting information file under the terms of the Creative Commons Attribution License (https://creativecommons.org/licenses/by/4.0). Please note that the reuse, redistribution and reproduction in particular requires that the author(s) and source are credited and that individual graphics may be subject to special legal provisions.
The license is subject to the *Beilstein Journal of Organic Chemistry* terms and conditions: (https://www.beilstein-journals.org/bjoc/terms)

Table of contents

Experimental procedures S4

General method of the synthesis nitroxides **2d,e** via reaction of nitrone with alkynylmagnesium bromide S4

2,2,5-Triethyl-3,4-bis(hydroxymethyl)-5-((trimethylsilyl)ethynyl)pyrrolidin-1-oxyl (**2d**) S5

2-(3-(Benzyloxy)prop-1-yn-1-yl)-2,5,5-triethyl-3,4-bis(hydroxymethyl)pyrrolidin-1-oxyl (**2e**) S5

2-(3-(Dimethylamino)prop-1-yn-1-yl)-2,5,5-triethyl-3,4-bis(hydroxymethyl)pyrrolidin-1-oxyl (**2f**) ... S6

General method of mesylation of nitroxides S6

2,2,5-Triethyl-5-ethynyl-3,4-bis(((methylsulfonyl)oxy)methyl)pyrrolidin-1-oxyl (**3a**) S7

2,2,5-Triethyl-3,4-bis(((methylsulfonyl)oxy)methyl)-5-(phenylethynyl)pyrrolidin-1-oxyl (**3b**) S7

2,2,5-Triethyl-3,4-bis(((methylsulfonyl)oxy)methyl)-5-(3-((methylsulfonyl)oxy)prop-1-yn-1-yl)pyrrolidin-1-oxyl (**3c**) S7

2,2,5-Triethyl-3,4-bis(((methylsulfonyl)oxy)methyl)-5-((trimethylsilyl)ethynyl)pyrrolidin-1-oxyl (**3d**) S8

2-(3-(Benzyloxy)prop-1-yn-1-yl)-2,5,5-triethyl-3,4-bis(((methylsulfonyl)oxy)methyl)pyrrolidin-1-oxyl (**3e**) S8

2-(3-(Dimethylamino)prop-1-yn-1-yl)-2,5,5-triethyl-3,4-bis(((methylsulfonyl)oxy)methyl)pyrrolidin-1-oxyl (**3f**) S8

General method for the synthesis of tricyclic fused triazole derivatives S9

6-(Azidomethyl)-3b,5,5-triethyl-3b,4,5,6,6a,7-hexahydropyrrolo[2',3':3,4]pyrrolo[1,2-*c*][1,2,3]triazol-4-oxyl (**4a**) S9

6-(Azidomethyl)-3b,5,5-triethyl-3-phenyl-3b,4,5,6,6a,7-hexahydropyrrolo[2',3':3,4]pyrrolo[1,2-*c*][1,2,3]triazol-4-oxyl (**4b**) S9

3,6-Bis(azidomethyl)-3b,5,5-triethyl-3b,4,5,6,6a,7-hexahydropyrrolo[2',3':3,4]pyrrolo[1,2-c][1,2,3]triazol-4-oxyl (**4c**) S9

6-(Azidomethyl)-3b,5,5-triethyl-3-(trimethylsilyl)-3b,4,5,6,6a,7-hexahydropyrrolo[2',3':3,4]pyrrolo[1,2-*c*][1,2,3]triazol-4-oxyl (**4d**) S10

6-(Azidomethyl)-3-((benzyloxy)methyl)-3b,5,5-triethyl-3b,4,5,6,6a,7-hexahydropyrrolo[2',3':3,4]pyrrolo[1,2-*c*][1,2,3]triazol-4-oxyl (**4e**) S10

6-(Azidomethyl)-3-((dimethylamino)methyl)-3b,5,5-triethyl-3b,4,5,6,6a,7-hexahydropyrrolo[2',3':3,4]pyrrolo[1,2-*c*][1,2,3]triazol-4-oxyl (**4f**) S10

3,4-Bis(acetoxymethyl)-2,2,5-triethyl-5-ethynylpyrrolidin-1-oxyl (**5**) S10

General method for the synthesis of alkynones S11

3,4-Bis(acetoxymethyl)-2,2,5-triethyl-5-(3-oxo-3-phenylprop-1-yn-1-yl)pyrrolidin-1-oxyl (**6a**) S11

3,4-Bis(((methylsulfonyl)oxy)methyl)-2,2,5-triethyl-5-(3-oxo-3-phenylprop-1-yn-1-yl)pyrrolidin-1-oxyl (**6b**) S11

2,2,6a-Triethyl-3-(hydroxymethyl)-1-6-(2-oxoethylidene)hexahydro-1*H*-furo[3,4-*b*]pyrrol-1-oxyl (**8**) S11

2,2,5-Triethyl-3,4-bis(hydroxymethyl)-5-(5-phenyl-1*H*-pyrazol-3-yl)pyrrolidin-1-oxyl (**9a**) S12

Annulated tricyclic pyrazole derivatives synthesis S12

3b,5,5-Triethyl-6-(((methylsulfonyl)oxy)methyl)-2-phenyl-3b,4,5,6,6a,7-hexahydropyrrolo[2',3':3,4]pyrrolo[1,2-*b*]pyrazol-4-oxyl (**9b**) S13

3b,5,5-Triethyl-6-(hydroxymethyl)-2-phenyl-3b,4,5,6,6a,7-hexahydropyrrolo[2',3':3,4]pyrrolo[1,2-*b*]pyrazol-4-oxyl (**9c**) S14

NMR spectra S15

Figure S1. ^{1}H NMR (300 MHz, CD_3OD, Zn/CF_3COOH) of 2,2,5-triethyl-3,4-bis(hydroxymethyl)-5-((trimethylsilyl)ethynyl)pyrrolidin-1-oxyl (**2d**) S15

Figure S2. ^{1}H NMR (300 MHz, CD_3OD, Zn/CF_3COOH) of 2-(3-(benzyloxy)prop-1-yn-1-yl)-2,5,5-triethyl-3,4-bis(hydroxymethyl)pyrrolidin-1-oxyl (**2e**) S15

Figure S3. ^{1}H NMR (500 MHz, CD_3OD, Zn/CF_3COOH) of 2-(3-(dimethylamino)prop-1-yn-1-yl)-2,5,5-triethyl-3,4-bis(hydroxymethyl)pyrrolidin-1-oxyl (**2f**) S16

Figure S4. ^{1}H NMR (300 MHz, CD_3OD, Zn/CF_3COOH) of 2,2,5-triethyl-5-ethynyl-3,4-bis(((methylsulfonyl)oxy)methyl)pyrrolidin-1-oxyl (**3a**) S16

Figure S5. ^{1}H NMR (300 MHz, CD_3OD, Zn/CF_3COOH) of 2,2,5-triethyl-3,4-bis(((methylsulfonyl)oxy)methyl)-5-(phenylethynyl)pyrrolidin-1-oxyl (**3b**) S17

Figure S6. ^{1}H NMR (300 MHz, CD_3OD, Zn/CF_3COOH) of 2,2,5-triethyl-3,4-bis(((methylsulfonyl)oxy)methyl)-5-((trimethylsilyl)ethynyl)pyrrolidin-1-oxyl (**3d**) S17

Figure S7. ^{1}H NMR (300 MHz, CD_3OD, Zn/CF_3COOH) of 2-(3-(dimethylamino)prop-1-yn-1-yl)-2,5,5-triethyl-3,4-bis(((methylsulfonyl)oxy)methyl)pyrrolidin-1-oxyl (**3e**) S18

Figure S8. ^{1}H NMR (400 MHz, CD_3OD, Zn/CF_3COOH) of 2-(3-(dimethylamino)prop-1-yn-1-yl)-2,5,5-triethyl-3,4-bis(((methylsulfonyl)oxy)methyl)pyrrolidin-1-oxyl (**3f**) S18

Figure S9. ^{1}H NMR (400 MHz, CD_3OD, Zn/CF_3COOH) of 3,4-bis(acetoxymethyl)-2,2,5-triethyl-5-ethynylpyrrolidin-1-oxyl (**5**) S19

Figure S10. ^{1}H NMR (300 MHz, CD_3OD, Zn/CF_3COOH) of 2,2,5-triethyl-3,4-bis(hydroxymethyl)-5-(3-phenyl-1*H*-pyrazol-5-yl)pyrrolidin-1-oxyl (**9a**) S19

Figure S11. ^{1}H NMR (300 MHz, CD_3OD, Zn/CF_3COOH) of 3b,5,5-triethyl-6-(((methylsulfonyl)oxy)methyl)-2-phenyl-3b,4,5,6,6a,7-hexahydropyrrolo[2',3':3,4]pyrrolo[1,2-*b*]pyrazol-4-oxyl (**9b**) S20

Figure S12. ^{1}H NMR (300 MHz, CD_3OD, Zn/CF_3COOH) of 3b,5,5-triethyl-6-(hydroxymethyl)-2-phenyl-3b,4,5,6,6a,7-hexahydropyrrolo[2',3':3,4]pyrrolo[1,2-*b*]pyrazol-4-oxyl (**9c**) S20

IR spectra S21

Figure S13. IR (KBr) of 2,2,5-triethyl-3,4-bis(hydroxymethyl)-5-((trimethylsilyl)ethynyl)pyrrolidin-1-oxyl (**2d**) S21

Figure S14. IR (neat) of 2-(3-(benzyloxy)prop-1-yn-1-yl)-2,5,5-triethyl-3,4-bis(hydroxymethyl)pyrrolidin-1-oxyl (**2e**) S21

Figure S15. IR (neat) of 2-(3-(dimethylamino)prop-1-yn-1-yl)-2,5,5-triethyl-3,4-bis(hydroxymethyl)pyrrolidin-1-oxyl (**2f**) S22

Figure S16. IR (KBr) of 2,2,5-triethyl-5-ethynyl-3,4-bis(((methylsulfonyl)oxy)methyl)pyrrolidin-1-oxyl (**3a**) S22

Figure S17. IR (neat) of 2,2,5-triethyl-3,4-bis(((methylsulfonyl)oxy)methyl)-5-(phenylethynyl)pyrrolidin-1-oxyl (**3b**) S22

Figure S18. IR (neat) of 2,2,5-triethyl-3,4-bis(((methylsulfonyl)oxy)methyl)-5-(3-((methylsulfonyl)oxy)prop-1-yn-1-yl)pyrrolidin-1-oxyl (**3c**)........ S23

Figure S19. IR (neat) of 2,2,5-triethyl-3,4-bis(((methylsulfonyl)oxy)methyl)-5-((trimethylsilyl)ethynyl)pyrrolidin-1-oxyl (**3d**)........ S23

Figure S20. IR (neat) of 2-(3-(benzyloxy)prop-1-yn-1-yl)-2,5,5-triethyl-3,4-bis(((methylsulfonyl)oxy)methyl)pyrrolidin-1-oxyl (**3e**) S23

Figure S21. IR (neat) of 2-(3-(dimethylamino)prop-1-yn-1-yl)-2,5,5-triethyl-3,4-bis(((methylsulfonyl)oxy)methyl)pyrrolidin-1-oxyl (**3f**)........ S24

Figure S22. IR (KBr) of 6-(azidomethyl)-3b,5,5-triethyl-3b,4,5,6,6a,7-hexahydropyrrolo[2',3':3,4]pyrrolo[1,2-*c*][1,2,3]triazol-4-oxyl (**4a**)........ S24

Figure S23. IR (KBr) of 6-(azidomethyl)-3b,5,5-triethyl-3-phenyl-3b,4,5,6,6a,7-hexahydropyrrolo[2',3':3,4]pyrrolo[1,2-*c*][1,2,3]triazol-4-oxyl (**4b**)........ S24

Figure S24. IR (KBr) of 3,6-bis(azidomethyl)-3b,5,5-triethyl-3b,4,5,6,6a,7-hexahydropyrrolo[2',3':3,4]pyrrolo[1,2-*c*][1,2,3]triazol-4-oxyl (**4c**) S25

Figure S25. IR (KBr) of 6-(azidomethyl)-3b,5,5-triethyl-3-(trimethylsilyl)-3b,4,5,6,6a,7-hexahydropyrrolo[2',3':3,4]pyrrolo[1,2-*c*][1,2,3]triazol-4-oxyl (**4d**)........ S25

Figure S26. IR (neat) of 6-(azidomethyl)-3-((benzyloxy)methyl)-3b,5,5-triethyl-3b,4,5,6,6a,7-hexahydropyrrolo[2',3':3,4]pyrrolo[1,2-*c*][1,2,3]triazol-4-oxyl (**4e**) S25

Figure S27. IR (neat) of 6-(azidomethyl)-3-((dimethylamino)methyl)-3b,5,5-triethyl-3b,4,5,6,6a,7-hexahydropyrrolo[2',3':3,4]pyrrolo[1,2-*c*][1,2,3]triazol-4-oxyl (**4f**). S26

Figure S28. IR (neat) of 3,4-bis(acetoxymethyl)-2,2,5-triethyl-5-ethynylpyrrolidin-1-oxyl (**5**) S26

Figure S29. IR (neat) of 3,4-bis(acetoxymethyl)-2,2,5-triethyl-5-(3-oxo-3-phenylprop-1-yn-1-yl)pyrrolidin-1-oxyl (**6a**)........ S26

Figure S30. IR (neat) of 2,2,5-triethyl-3,4-bis(((methylsulfonyl)oxy)methyl)-5-(3-oxo-3-phenylprop-1-yn-1-yl)pyrrolidin-1- oxyl (**6b**)........ S27

Figure S31. IR (KBr) of (Z)-2,2,6a-triethyl-3-(hydroxymethyl)-6-(2-oxoethylidene)hexahydro-1*H*-furo[3,4-*b*]pyrrol-1-oxyl (**8**) S27

Figure S32. IR (neat) 2,2,5-triethyl-3,4-bis(hydroxymethyl)-5-(3-phenyl-1*H*-pyrazol-5-yl)pyrrolidin-1-oxyl (**9a**)........ S27

Figure S33. IR (neat) 3b,5,5-triethyl-6-(((methylsulfonyl)oxy)methyl)-2-phenyl-3b,4,5,6,6a,7-hexahydropyrrolo[2',3':3,4]pyrrolo[1,2-*b*]pyrazol-4-oxyl (**9b**) S28

Figure S34. IR (KBr) 3b,5,5-triethyl-6-(hydroxymethyl)-2-phenyl-3b,4,5,6,6a,7-hexahydropyrrolo[2',3':3,4]pyrrolo[1,2-*b*]pyrazol-4-oxyl (**9c**)........ S28

EPR measurements........ S29

X-ray crystallographic analysis........ S37

Experimental procedures

All commercially available solvents and reagents were of highest quality and used without further purification. Nitrone **1** and nitroxides **2a–c** was prepared according to the literature protocols [1]. NMR spectra for nitroxides were recorded after reduction by the Zn/CF_3COOH system in CD_3OD as describe [S1,S2]. ^{1}H NMR spectra were recorded at 300, 400 or 500 MHz on Bruker Avance AV 300, AV 400 and DRX 500 spectrometers, as indicated next to each NMR analysis. ^{1}H chemical shifts (δ) were internally referenced to the residual solvent peak. The *J* values in ^{1}H NMR spectra are given in Hz. The IR spectra were acquired on a FTIR spectrometer in KBr or neat. The UV spectra were recorded in EtOH (10^{-4} M solutions). The progress of the reactions was monitored by TLC and carried out using UV light at 254 nm, 10% solution of phosphomolybdic acid in ethanol, and/or Dragendorff reagent as visualizing agents. Column chromatography was performed on silica gel 60 (70−230 mesh). HRMS were recorded on a double-focusing, high-resolution mass spectrometer equipped with a high-performance toroidal ESA and quadrupole time-of-flight mass spectrometer\

S1. Dobrynin, S. A.; Gulman, M. M.; Morozov, D. A.; Zhurko, I. F.; Taratayko, A. I.; Sotnikova, Y. S.; Glazachev, Y. I.; Gatilov, Y. V.; Kirilyuk, I. A. Synthesis of Sterically Shielded Nitroxides Using the Reaction of Nitrones with Alkynylmagnesium Bromides. *Molecules* **2022**, 27 (21), 7626–7626. https://doi.org/10.3390/molecules27217626.

S2. Dobrynin, S. A.; Usatov, M. S.; Zhurko, I. F.; Morozov, D. A.; Polienko, Y. F.; Glazachev, Y. I.; Parkhomenko, D. A.; Tyumentsev, M. A.; Gatilov, Y. V.; Chernyak, E. I.; Bagryanskaya, E. G.; Kirilyuk, I. A. A Simple Method of Synthesis of 3-Carboxy-2,2,5,5-Tetraethylpyrrolidine-1-Oxyl and Preparation of Reduction-Resistant Spin Labels and Probes of Pyrrolidine Series. *Molecules* **2021**, 26 (19), 5761–5761. https://doi.org/10.3390/molecules26195761.

General method of the synthesis nitroxides 2d,e via reaction of nitrone with alkynylmagnesium bromide

A solution of corresponding terminal alkyne (trimethylsilylacetylene or benzyl propargyl ether) (0.14 mol) in dry THF (30 mL) was added dropwise to a solution of EtMgBr (2 M in THF, 70 mL). The mixture was stirred at room temperature for 30 min. A solution of nitrone **1** (5 g, 13.4 mmol) in dry THF (50 mL) was added to reaction mixture, and stirred for 3 days at room temperature, monitored by TLC (SiO_2, MeOH:ethyl acetate = 1:9, UV light for visualization). Upon completion, reaction mixture was quenched with water (10–15 mL). The precipitate was washed with ether (4 × 15 mL), and the combined organic phase was evaporated under reduced pressure The residue was dissolved in a mixture of methanol (50 mL) and water (10 mL).

Pyridinium *p*-toluenesulfonate (2 g, 8 mmol) was then added. The mixture stirred in air for oxidation (8 h). The reaction was monitored by TLC (SiO_2, MeOH:ethyl acetate = 1:9, Dragendorff reagent for visualization). After deprotection and oxidation, the reaction mixture was basified with 10 mL of 1 M aqueous solution of Na_2CO_3, and the methanol was removed in vacuum. The aqueous layer was extracted with ether (3 × 15 mL), dried over (Na_2CO_3), and evaporated in vacuum. The residue was purified by column chromatography (SiO_2, mixture of hexane and ethyl acetate).

2,2,5-Triethyl-3,4-bis(hydroxymethyl)-5-((trimethylsilyl)ethynyl)pyrrolidin-1-oxyl (2d)

Yield 2.9 g (64%), yellow crystals, m.p. 69-74 °C. IR (KBr) ν_{max} (cm^{-1}): 3473 (O-H), 3363 (O-H), 2968 (C-H), 2941 (C-H), 2881 (C-H), (C-H). 2171 (C≡C), 1247 (Si-CH_3); Anal. Calcd for $C_{17}H_{32}NO_3Si$: C, 62.53; H, 9.88; N, 4.29; SiO_2, 18.40; found: C, 62.40; H, 9.98; N, 4.57. SiO_2, 18.45; HRMS (EI/DFS) m/z $[M]^+$ Calcd. for $C_{17}H_{32}NO_3{}^{28}Si$: 326.2146; found: 326.2141; ^{1}H NMR (300 MHz, CD_3OD, Zn/CF_3COOH, δ (ppm)): 0.21 (s, 9H), 0.97 (t, J_t=7.4 Hz, 3H), 0.97 (t, J_t=7.4 Hz, 3H), 1.19 (t, J_t=7.2 Hz, 3H), 1.85 (dq, J_d=-14.0 Hz, J_q=7.4 Hz, 1H), 1.94 (dq, J_d=-14.0 Hz, J_q=7.4 Hz, 1H), 2.00 (dq, J_d=-13.6 Hz, J_q=7.4 Hz, 1H), 2.11 (dq, J_d=-13.6 Hz, J_q=7.4 Hz, 1H), 2.14 (dq, J_d=-13.2 Hz, J_q=7.2 Hz, 1H), 2.23 (dq, J_d=-13.2 Hz, J_q=7.2 Hz, 1H), 2.29 (dd, J_{d1}=2.3 Hz, J_{d2}=2.3 Hz, 1H), 3.29 (ddd, J_{d1}=6.0 Hz, J_{d2}=4.2 Hz, J_{d3}=4.2 Hz, 1H), 3.71 (d, J_d=4.2 Hz, 2H), 3.81 (dd, J_{d1}=2.3 Hz, J_{d2}=-12.1 Hz, 1H), 3.89 (dd, J_{d1}=2.3 Hz, J_{d2}=-12.1 Hz 1H).

2-(3-(Benzyloxy)prop-1-yn-1-yl)-2,5,5-triethyl-3,4-bis(hydroxymethyl)pyrrolidin-1-oxyl (2e)

Yield 3.3g (66%), yellow oil, IR (neat) ν_{max} (cm^{-1}): 3375 (O-H), 3089 (=C-H), 3064 (=C-H), 3031 (=C-H), 2970 (C-H), 2939 (C-H), 2881 (C-H). Anal. Calcd for $C_{22}H_{32}NO_4$: C, 70.56; H, 8.61; N, 3.74; Found: C, 70.70; H, 8.84; N, 3.46. HRMS (EI/DFS) m/z $[M]^+$ Calcd. for $C_{22}H_{32}NO_4$: 374.2326; found: 374.2324. ^{1}H NMR (300 MHz, CD_3OD, Zn/CF_3COOH, δ (ppm)): 0.96 (t, J_t=7.5 Hz, 3H), 0.99 (t, J_t=7.4 Hz, 3H), 1.21 (t, J_t=7.1 Hz, 3H), 1.89 (dq, J_d=-14.7 Hz, J_q=7.5 Hz, 1H), 1.97 (dq, J_d=-14.2 Hz, J_q=7.1 Hz, 1H), 2.03 (dq, J_d=-14.2 Hz, J_q=7.2 Hz, 1H), 2.10 (dq, J_d=-14.0 Hz, J_q=7.4 Hz, 1H), 2.13 (dq, J_d=-14.0 Hz, J_q=7.4 Hz, 1H), 2.28 (dq, J_d=-14.7 Hz, J_q=7.5 Hz, 1H), 2.28 (ddd, J_{d1}=4.3 Hz, J_{d2}=4.7 Hz, J_{d3}=9.9 Hz, 1H), 2.34 (ddd, J_{d1}=5.8 Hz, J_{d2}=7.0 Hz, J_{d3}=9.9 Hz, 1H), 3.72 (d, J_d=4.7 Hz, 2H), 3.85 (dd, J_{d1}=5.8 Hz, J_{d2}=-11.5 Hz, 1H), 3.90 (dd, J_{d1}=7.0 Hz, J_{d2}=-11.5 Hz, 1H), 4.33 (s, 2H), 4.62 (s, 2H); 7.30-7.40 (m, 5H).

2-(3-(Dimethylamino)prop-1-yn-1-yl)-2,5,5-triethyl-3,4-bis(hydroxymethyl)pyrrolidin-1-oxyl (2f)

Copper(II) acetate monohydrate (0.2 g, 1 mmol) was added to a mixture of nitroxide **2a** (4.0 g, 15.7 mmol), 20% aqueous solution of formaldehyde (9.3 g, 62 mmol), 33% aqueous solution of dimethylamine (6.4 g, 47 mmol) in THF (350 mL) and heated under reflux for 24 h. The reaction was monitored by TLC (SiO_2, MeOH:ethyl acetate = 1:9, UV light for visualization). After cooling, the mixture was diluted with water (100 mL), and the THF was removed under reduced pressure. The resulting aqueous suspension was extracted with EtOAc (4 × 50 mL). The combined organic extracts were washed with water (50 mL) and then back-extracted with a 1 M aqueous solution of monosodium phosphate (2 × 100 mL). The combined water extract was basified with Na_2CO_3 to pH > 11 and extracted with ethyl acetate. The organic phase was dried over Na_2CO_3, filtered and concentrated under reduced pressure. The residue was purified by column chromatography (SiO_2, hexane:ethyl acetate = 2:1).

Yield 4.67 g (95%), orange oil. IR (neat) ν_{max} (cm^{-1}): 3367 (O-H), 2968 (C-H), 2937 (C-H), 2879 (C-H), 2780 (C-H), 1043 (C-O). HRMS (EI/DFS) m/z [M]+ Calcd. for $C_{17}H_{31}N_2O_3$: 311.2379; found: 311.2382. ^1H NMR (500 MHz, CD_3OD, Zn/CF_3COOH, δ (ppm)): 0.98 (t, J_t=7.4 Hz, 3H), 1.02 (t, J_t=7.4 Hz, 3H), 1.21 (t, J_t=7.4 Hz, 3H), 1.97 (dq, J_d=-15.1 Hz, J_q=7.4 Hz, 1H), 1.98 (dq, J_d=-15.1 Hz, J_q=7.4 Hz, 1H), 2.06 (dq, J_d=-14.7 Hz, J_q=7.4 Hz, 1H), 2.08 (dq, J_d=-9.8 Hz, J_q=7.4 Hz, 1H), 2.11 (dq, J_d=-9.8 Hz, J_q=7.4 Hz, 1H), 2.32 (dq, J_d=-14.7 Hz, J_q=7.4 Hz, 1H), 2.30 (ddd, J_{d1}=4.8 Hz, J_{d2}=5.1 Hz, J_{d3}=9.6 Hz, 1H), 2.46 (ddd, J_{d1}=6.0 Hz, J_{d2}=6.9 Hz, J_{d3}=9.4 Hz, 1H), 3.01 (s, 6H); 3.73 (d, J_d=4.8 Hz, 1H), 3.73 (d, J_d=5.1 Hz, 1H), 3.89 (d, J_d=6.0 Hz, 1H), 3.89 (d, J_d=6.9 Hz, 1H), 4.28 (s, 2H).

General method of mesylation of nitroxides

A mixture of corresponding nitroxide **2a–f*** (2 mmol), DIPEA (6 mmol), MsCl (5 mmol) and dry chloroform (50 mL) was heated under reflux for 30 min. The reaction was monitored by TLC (SiO_2, mixtures of hexane and ether, 10% solution of phosphomolybdic acid for visualization). The mixture was washed with hot water. The organic layer was dried over Na_2CO_3, filtered and concentrated under reduced pressure. The residue was purified by column chromatography (SiO_2, hexane: ethyl acetate).

* Note: For nitroxide **2c**, the amounts of reagents were adjusted: DIPEA (8.5 mmol) and MsCl (7.5 mmol) were used.

2,2,5-Triethyl-5-ethynyl-3,4-bis(((methylsulfonyl)oxy)methyl)pyrrolidin-1-oxyl (3a)

Yield (91%) from compound **2a**, yellow crystals, m.p. 72-76 °C. IR (KBr) ν_{max} (cm^{-1}): 3272 (≡C-H), 3031 (C-H), 2972 (C-H), 2941 (C-H), 2881 (C-H), 2112 (C≡C), 1357 (S=O), 1174 (S=O). Anal. Calcd for $C_{16}H_{28}NO_7S_2$: C, 46.81; H, 6.87; N, 3.41; S, 15.62; found: C, 47.06; H, 6.73; N, 3.53; S, 15.57. HRMS (EI/DFS) m/z $[M]^+$ Calcd for $C_{16}H_{28}NO_7{}^{32}S_2$: 410.1300; found: 410.1302. ^1H NMR (300 MHz, CD_3OD, Zn/CF_3COOH, δ (ppm)): 0.99 (t, J_t=7.4 Hz, 3H), 1.02 (t, J_t=7.3 Hz, 3H), 1.23 (t, J_t=7.4 Hz, 3H), 1.92 (dq, J_d=-15.0 Hz, J_q=7.3 Hz, 1H), 1.97 (dq, J_d=-15.0 Hz, J_q=7.3 Hz, 1H), 2.07 (dq, J_d=-14.6 Hz, J_q=7.4 Hz, 1H), 2.17 (dq, J_d=-13.5 Hz, J_q=7.4 Hz, 1H), 2.21 (dq, J_d=-13.5 Hz, J_q=7.4 Hz, 1H), 2.22 (dq, J_d=-14.6 Hz, J_q=7.4 Hz, 1H), 2.73 (ddd, J_{d1}=3.7 Hz, J_{d2}=5.5 Hz, J_{d3}=10.8 Hz, 1H), 2.75 (ddd, J_{d1}=4.7 Hz, J_{d2}=7.2 Hz, J_{d3}=10.8 Hz, 1H), 3.16 (s, 3H), 3.17 (s, 3H), 3.65 (s, 1H), 4.42 (dd, J_{d1}=3.8 Hz, J_{d2}=-11.3 Hz, 1H), 4.51 (dd, J_{d1}=5.5 Hz, J_{d2}=-11.3 Hz, 1H), 4.58 (dd, J_{d1}=7.2 Hz, J_{d2}=-11.0 Hz, 1H), 4.60 (dd, J_{d1}=4.7 Hz, J_{d2}=-11.0 Hz, 1H);

2,2,5-Triethyl-3,4-bis(((methylsulfonyl)oxy)methyl)-5-(phenylethynyl)pyrrolidin-1-oxyl (3b)

Yield (84%) from compound **2b**, yellow oil. IR (neat) ν_{max} (cm^{-1}): 3024 (=C-H), 2974 (C-H), 2939 (C-H), 2883 (C-H), 1358 (S=O), 1176 (S=O). Anal. Calcd for $C_{22}H_{32}NO_7S_2$: C, 54.30; H, 6.63; N, 2.88; S, 13.18; Found: C, 53.97; H, 6.69; N, 2.80; S, 13.42. HRMS (EI/DFS) m/z $[M]^+$ Calcd for $C_{22}H_{32}NO_7{}^{32}S_2$: 489.1615; found: 489.1609. ^1H NMR (300 MHz, CD_3OD, Zn/CF_3COOH, δ (ppm)): 1.00 (t, J_t=7.3 Hz, 3H), 1.05 (t, J_t=7.1 Hz, 3H), 1.30 (t, J_t=7.3 Hz, 3H), 1.99 (q, J_q=7.3 Hz, 2H), 2.18 (dq, J_d=-14.1 Hz, J_q=7.3 Hz, 1H), 2.26 (dq, J_d=-14.1 Hz, J_q=7.1 Hz, 1H), 2.28 (dq, J_d=-14.1 Hz, J_q=7.1 Hz, 1H), 2.31 (dq, J_d=-14.5 Hz, J_q=7.3 Hz, 1H), 2.82 (ddd, J_{d1}=4.4 Hz, J_{d2}=5.7 Hz, J_{d3}=10.0 Hz, 1H), 2.83 (ddd, J_{d1}=4.3 Hz, J_{d2}=7.0 Hz, J_{d3}=10.0 Hz, 1H), 3.09 (s, 3H), 3.14 (s, 3H), 4.45 (dd, J_{d1}=4.4 Hz, J_{d2}=-11.1 Hz, 1H), 4.54 (dd, J_{d1}=5.7 Hz, J_{d2}=-11.1 Hz, 1H), 4.64 (dd, J_{d1}=4.3 Hz, J_{d2}=-10.7 Hz, 1H), 4.67 (dd, J_{d1}=7.0 Hz, J_{d2}=-10.7 Hz, 1H), 7.36 (dddd, J_{d1}=0.8 Hz, J_{d2}=1.6 Hz, J_{d3}=7.6 Hz, J_{d4}=7.7 Hz, 1H), 7.36 (dddd, J_{d1}=0.8 Hz, J_{d2}=1.6 Hz, J_{d3}=7.6 Hz, J_{d4}=7.7 Hz, 1H), 7.41 (dddd, J_{d1}=1.3 Hz, J_{d2}=1.3 Hz, J_{d3}=7.6 Hz, J_{d4}=7.6 Hz, 1H), 7.52 (dddd, J_{d1}=0.8 Hz, J_{d2}=1.3 Hz, J_{d3}=2.2 Hz, J_{d4}=7.7 Hz, 1H), 7.52 (dddd, J_{d1}=0.8 Hz, J_{d2}=1.3 Hz, J_{d3}=2.2 Hz, J_{d4}=7.7 Hz, 1H).

2,2,5-Triethyl-3,4-bis(((methylsulfonyl)oxy)methyl)-5-(3-((methylsulfonyl)oxy)prop-1-yn-1-yl)pyrrolidin-1-oxyl (3c)

Yield (83%) from compound **2c**, yellow oil. IR (neat) ν_{max} (cm^{-1}): 2976 (C-H), 2941 (C-H), 2885 (C-H), 1354 (S=O), 1176 (S=O). Anal. Calcd for $C_{18}H_{32}NO_{10}S_3$: C, 41.69; H, 6.22; N, 2.70; S, 18.54, found: C, 41.35; H, 6.34; N, 2.57; S, 18.63.

2,2,5-Triethyl-3,4-bis(((methylsulfonyl)oxy)methyl)-5-((trimethylsilyl)ethynyl)pyrrolidin-1-oxyl (3d)

Yield (91%) from compound **2d**, yellow oil. IR (neat) ν_{max} (cm^{-1}): 2970 (C-H), 2941 (C-H), 2902 (C-H), 2885 (C-H), 2165 (C≡C), 1357 (S=O), 1178 (S=O). Anal. Calcd for $C_{19}H_{36}NO_7S_2Si$: C, 47.28; H, 7.52; N, 2.90; S, 13.29; Found: C, 47.52; H, 7.50; N, 3.05; S, 13.65. HRMS(EI/DFS) m/z $[M]^+$ Calcd for $C_{19}H_{36}NO_7{}^{32}S_2Si$: 482.1697, found: 482.1700. ^1H NMR (300 MHz, CD_3OD, Zn/CF_3COOH, δ (ppm)): 0.24 (s, 9H), 0.99 (t, J_t=7.4 Hz, 3H), 1.01 (t, J_t=7.3 Hz, 3H), 1.22 (t, J_t=7.4 Hz, 3H), 1.93 (dq, J_d=-16.6 Hz, J_q=7.3 Hz, 1H), 1.93 (dq, J_d=-15.8 Hz, J_q=7.3 Hz, 1H), 2.03 (dq, J_d=-14.3 Hz, J_q=7.4 Hz, 1H), 2.18 (dq, J_d=-16.6 Hz, J_q=7.3 Hz, 1H), 2.20 (dq, J_d=-14.3 Hz, J_q=7.4 Hz, 1H), 2.21 (dq, J_d=-15.8 Hz, J_q=7.4 Hz, 1H), 2.70 (ddd, J_{d1}=4.1 Hz, J_{d2}=6.0 Hz, J_{d3}=10.9 Hz, 1H), 2.71 (ddd, J_{d1}=3.7 Hz, J_{d2}=7.8 Hz, J_{d3}=10.9 Hz, 1H), 3.14 (s, 3H), 3.15 (s, 3H), 4.40 (dd, J_{d1}=4.1 Hz, J_{d2}=-11.2 Hz, 1H), 4.50 (dd, J_{d1}=6.0 Hz, J_{d2}=-11.2 Hz, 1H), 4.56 (dd, J_{d1}=7.8 Hz, J_{d2}=-12.4 Hz, 1H), 4.57 (dd, J_{d1}=3.7 Hz, J_{d2}=-12.4 Hz, 1H).

2-(3-(Benzyloxy)prop-1-yn-1-yl)-2,5,5-triethyl-3,4-bis(((methylsulfonyl)oxy)methyl)pyrrolidin-1-oxyl (3e)

Yield (75%) from **2e**, yellow oil. IR (neat) ν_{max} (cm^{-1}): 3027 (C-H), 2972 (C-H), 2939 (C-H), 2883 (C-H), 1358 (S=O), 1176 (S=O). Anal. Calcd for $C_{24}H_{36}O_8NS_2$: C, 54.32; H, 6.84; N, 2.64; S, 12.08, found: C, 54.52; H, 6.99; N, 2.78; S, 11.58. HRMS (EI/DFS) m/z $[M]^+$ Calcd for $C_{24}H_{36}O_8N^{32}S_2$: 530.1877; found: 530.1878. ^1H NMR (400 MHz, CD_3OD, Zn/CF_3COOH, δ (ppm)): 0.62 (t, J_t=7.5 Hz, 3H), 0.81 (t, J_t=7.4 Hz, 3H), 1.34 (t, J_t=7.4 Hz, 3H), 1.14-1.77 (m, 6H), 2.07-2.23 (m, 2H), 2.78 (s, 3H), 2.81 (s, 3H), 3.97 (s, 2H), 4.25 (s, 2H), 4.03 (dd, J_{d1}=5.1 Hz, J_{d2}=-10.1 Hz, 1H), 4.11 (dd, J_{d1}=5.0 Hz, J_{d2}=-10.1 Hz, 1H), 4.18 (dd, J_{d1}=5.3 Hz, J_{d2}=-10.1 Hz, 1H), 4.21 (dd, J_{d1}=5.7 Hz, J_{d2}=-10.1 Hz, 1H), 7.00-7.10 (m, 5H).

2-(3-(Dimethylamino)prop-1-yn-1-yl)-2,5,5-triethyl-3,4-bis(((methylsulfonyl)oxy)methyl)pyrrolidin-1-oxyl (3f)

Yield (77%) from compound **2f**, yellow oil. IR (neat) ν_{max} (cm^{-1}): 2974 (C-H), 2941 (C-H), 2988 (C-H), 2883 (C-H), 2825 (C-H), 2779 (C-H), 1356 (S=O), 1176 (S=O). Anal. Calcd for $C_{19}H_{35}O_7NS_2$: C, 48.80; H, 7.54; N, 5.99; S, 13.71, found: C, 49.22; H, 7.54; N, 5.77; S, 14.10. HRMS (EI/DFS) m/z $[M+1]^+$ Calcd for $C_{19}H_{36}O_7N^{32}S_2$: 468.1958; found: 468.1957. ^1H NMR (400 MHz, CD_3OD, Zn/CF_3COOH, δ (ppm)): 1.01 (t, J_t=7.3 Hz, 3H), 1.05 (t, J_t=7.2 Hz, 3H), 1.25 (t, J_t=7.3 Hz, 3H), 1.96 (dq, J_d=-15.3 Hz, J_q=7.2 Hz, 1H), 1.99 (dq, J_d=-15.3 Hz, J_q=7.2 Hz, 1H), 2.08 (dq, J_d=-14.6 Hz, J_q=7.3 Hz, 1H), 2.16 (dq, J_d=-14.6 Hz, J_q=7.3 Hz, 1H), 2.19 (dq, J_d=-15.2 Hz, J_q=7.3 Hz, 1H), 2.29 (dq, J_d=-15.2 Hz, J_q=7.3 Hz, 1H), 2.79 (ddd, J_{d1}=4.7 Hz, J_{d2} =5.7 Hz, J_{d3}=10.8 Hz, 1H), 2.81 (ddd, J_{d1}=5.7 Hz, J_{d2} =6.5 Hz, J_{d3}=10.8 Hz, 1H), 3.01 (s, 3H),

3.19 (s, 3H), 4.30 (d, J_d=-16.4 Hz, 1H), 4.32 (d, J_d=-16.4 Hz, 1H), 4.43 (dd, J_{d1}=4.7 Hz, J_{d2}=-11.3 Hz, 1H), 4.53 (dd, J_{d1}=5.7 Hz, J_{d2}=-11.3 Hz, 1H), 4.62 (dd, J_{d1}=5.7 Hz, J_{d2}=-11.7 Hz, 1H), 4.64 (dd, J_{d1}=6.4 Hz, J_{d2}=-11,7 Hz, 1H).

General method for the synthesis of tricyclic fused triazole derivatives

A mixture of corresponding nitroxide **3a–f*** (1mmol), sodium azide (4 mmol) and DMSO (10 mL) was stirred at 60 °C for 8 hours. The reaction was monitored by TLC (SiO_2, hexane:ethyl acetate mixtures, UV light for visualization). After cooling to room temperature, the mixture was diluted with water (50 mL) and extracted with ether (3 × 20 mL). The combined organic phase was evaporated under reduced pressure. The residue was purified by column chromatography (SiO_2, hexane:ethyl acetate mixtures).

* Note: For nitroxide **3c** 6 mmol of sodium azide was used.

6-(Azidomethyl)-3b,5,5-triethyl-3b,4,5,6,6a,7-hexahydropyrrolo[2',3':3,4]pyrrolo[1,2-*c*][1,2,3]triazol-4-oxyl (4a)

Yield (97%) from compound **3a**, yellow crystals, m.p. 126-128 °C. IR(KBr) ν_{max} (cm^{-1}): 2966 (C-H), 2879 (C-H), 2102 (N≡N). Anal. Calcd for $C_{14}H_{22}N_7O$: C, 55.25; H, 7.29; N, 32.21; found: C, 55.17; H, 7.29; N, 32.57. HRMS (EI/DFS) m/z $[M]^+$ Calcd for $C_{14}H_{22}N_7O$: 304.1880; found: 304.1884.

6-(Azidomethyl)-3b,5,5-triethyl-3-phenyl-3b,4,5,6,6a,7-hexahydropyrrolo[2',3':3,4]pyrrolo[1,2-*c*][1,2,3]triazol-4-oxyl (4b)

Yield (62%) from compound **3b**, yellow crystals, m.p. 108-109 °C. IR(KBr) ν_{max} (cm^{-1}): 3063 (=C-H), 3034 (=C-H), 2983 (C-H), 2980 (C-H) ,2937 (C-H), 2879 (C-H), 2112 (N≡N). Anal. Calcd for $C_{20}H_{26}N_7O$: C, 63.14; H, 6.89; N, 25.77; found: C, 63.03; H, 6.87; N, 25.78. HRMS (EI/DFS) m/z $[M]^+$ Calcd for $C_{20}H_{26}N_7O$: 380.2193; found: 380.2190.

3,6-Bis(azidomethyl)-3b,5,5-triethyl-3b,4,5,6,6a,7-hexahydropyrrolo[2',3':3,4]pyrrolo[1,2-c][1,2,3]triazol-4-oxyl (4c)

Yield (65%) from compound **3c**. yellow crystals, m.p. 85-86 °C. IR(KBr) ν_{max} (cm^{-1}): 2976 (C-H), 2964 (C-H), 2937 (C-H), 2924 (C-H), 2881 (C-H), 2116 (N=N=N), 2081 (N=N=N). Anal. Calcd for $C_{15}H_{23}N_{10}O$: C, 50.13; H, 6.45; N, 38.97; found: C, 50.38; H, 6.44; N, 38.95. HRMS(EI/DFS) m/z $[M]^+$ Calcd for $C_{15}H_{23}N_{10}O$: 359.2051; found: 359.2045.

6-(Azidomethyl)-3b,5,5-triethyl-3-(trimethylsilyl)-3b,4,5,6,6a,7-hexahydropyrrolo[2',3':3,4]pyrrolo[1,2-*c*][1,2,3]triazol-4-oxyl (4d)

Yield (45%) from **3d**, yellow crystals, m.p. 65-70 °C. IR(KBr) ν_{max} (cm^{-1}): 2968 (C-H), 2935 (C-H), 2877 (C-H), 2104 (N≡N). Anal. Calcd for $C_{17}H_{30}N_7OSi$: C, 54.22; H, 8.03; N, 26.04; found: C, 53.88; H, 8.08; N, 26.30. HRMS (EI/DFS) m/z $[M]^+$ Calcd for $C_{17}H_{30}N_7OSi$: 376.2276; found: 376.2271.

6-(Azidomethyl)-3-((benzyloxy)methyl)-3b,5,5-triethyl-3b,4,5,6,6a,7-hexahydropyrrolo[2',3':3,4]pyrrolo[1,2-*c*][1,2,3]triazol-4-oxyl (4e)

Yield (65%) from **3e**. yellow oil. IR(neat) ν_{max} (cm^{-1}): 3087 (=C-H), 3064 (=C-H), 3032 (=C-H), 2972 (C-H), 2937 (C-H), 2937 (C-H), 2881 (C-H), 2102 (N≡N). Anal. Calcd for $C_{22}H_{30}N_7O_2$: C, 62.24; H, 7.12; N, 23.10; found: C, 62.03; H, 6.87; N, 22.78. HRMS (EI/DFS) m/z $[M]^+$ Calcd for $C_{22}H_{30}N_7O_2$: 424.2456; found : 424.2446.

6-(Azidomethyl)-3-((dimethylamino)methyl)-3b,5,5-triethyl-3b,4,5,6,6a,7-hexahydropyrrolo[2',3':3,4]pyrrolo[1,2-*c*][1,2,3]triazol-4-oxyl (4f)

Yield (66%) from compound **3f**. yellow oil. IR(neat) ν_{max} (cm^{-1}): 2972 (C-H), 2939 (C-H), 2881 (C-H), 2823 (C-H), 2777 (C-H), 2100 (N≡N). Anal. Calcd for $C_{17}H_{29}N_8O$: C, 56.49; H, 8.09; N, 31.00. found: C, 56.49; H, 8.06; N, 30.82. HRMS (EI/DFS) m/z $[M]^+$ Calcd for $C_{17}H_{29}N_8O$: 361.2459; found: 361.2456.

3,4-Bis(acetoxymethyl)-2,2,5-triethyl-5-ethynylpyrrolidin-1-oxyl (5)

A mixture of nitroxide **2a** (1.5 g, 6 mmol), acetic anhydride (15 mL, 150 mmol), and sodium acetate (2.0 g, 25 mmol) in chloroform (15 mL) was stirred for 24 hours at room temperature. The reaction progress was monitored by TLC (SiO_2, hexane/ethyl acetate = 1:1, UV light for visualization). The precipitate was removed by filtration, and the filtrate was concentrated under reduced pressure. The residue was separated using column chromatography (SiO_2, hexane:ethyl acetate = 1:1) to afford the product as a yellow oil 1.7 g (84%). IR (neat) ν_{max} (cm^{-1}): 3259 (≡C-H), 2972 (C-H), 2939 (C-H), 2883 (C-H), 2107 (C≡C), 1743 (C=O), 1241 (C(O)-O), 1039 (C-O). Anal. Calcd. for $C_{18}H_{28}O_5N$: C, 63.88; H, 8.34; N, 4.14; found: C, 63.94; H, 8.40; N, 4.15; HRMS (EI/DFS) m/z $[M]^+$ Calcd. for $C_{18}H_{28}O_5N$: 338.1962; found: 338.1961; ^{1}H NMR (300 MHz, CD_3OD, Zn/CF_3COOH, δ) 0.98 (t, J_t=7.3 Hz, 3H), 1.01 (t, J_t=7.2 Hz, 3H), 1.23 (t, J_t=7.3 Hz, 3H); 1.90 (q, J_q=7.3 Hz, 2H), 2.06 (q, J_q=7.3 Hz, 2H), 2.19 (q, J_q=7.2 Hz, 2H); 2.05 (s, 3H), 2.07 (s, 3H); 2.53 (ddd, J_{d1}=4.7 Hz, J_{d2}=7.0 Hz, J_{d3}=11.4 Hz, 1H), 2.62 (ddd, J_{d1}=4.1 Hz, J_{d2}=6.4 Hz, J_{d3}=11.4 Hz, 1H); 3.48 (s, 1H); 4.26 (dd, J_{d1}=4.1 Hz, J_{d2}=-12.2 Hz, 1H),

4.32 (dd, J_{d1}=6.4 Hz, J_{d2}=-12.2 Hz, 1H), 4.34 (dd, J_{d}=7.0 Hz, J_{d2}=-11.9 Hz, 1H), 4.48 (dd, J_{d1}=4.7 Hz, J_{d2}=-11.9 Hz, 1H).

General method for the synthesis of alkynones

The reaction was performed under an argon atmosphere. To a stirred mixture of terminal alkyne (**5** or **3a**) (0.6 mmol), $Pd(PPh_3)_2Cl_2$ (17 mg, 0.024 mmol), CuI (5 mg, 0.024 mmol), PPh_3 (6 mg, 0.024 mmol), and NEt_3 (182 mg, 1.8 mmol) in toluene (15 mL) was added benzoyl chloride (168 mg, 1.2 mmol). The solution was heated under reflux for 4 hours, and monitored by TLC (SiO_2, hexane/ethyl acetate = 3:1, UV light for visualization). The solvent was evaporated under reduced pressure, and the residue was taken up in diethyl ether. The precipitate was removed by filtration, and the filtrate was concentrated under reduced pressure. The crude product was separated using column chromatography (SiO_2, hexane:ethyl acetate = 2:1, UV light for visualization) to afford the title compound.

3,4-Bis(acetoxymethyl)-2,2,5-triethyl-5-(3-oxo-3-phenylprop-1-yn-1-yl)pyrrolidin-1-oxyl (6a)

Yield 230 mg, (89%) from **5**. IR (neat) ν_{max} (cm^{-1}): 3026 (=C-H), 2974 (C-H), 2939 (C-H), 2883 (C-H), 2214 (C≡C), 1645 (C=O), 1265 (C(O)-O), 1358, 1176, 958, 831(C-O); Anal. Calcd. for $C_{25}H_{32}O_6N$: C, 67.85; H, 7.29; N, 3.17 found: C, 67.42; H, 7.17; N, 3.25; HRMS (EI/DFS) m/z $[M]^+$ Calcd. for $C_{25}H_{32}O_6N$: 442.2224; found: 442.2223.

3,4-Bis(((methylsulfonyl)oxy)methyl)-2,2,5-triethyl-5-(3-oxo-3-phenylprop-1-yn-1-yl)pyrrolidin-1-oxyl (6b)

Yield 136 mg (44%) from compound **3a**. yellow oil. IR (neat) ν_{max} (cm^{-1}): 3064 (=C-H), 2974 (C-H), 2939 (C-H), 2883 (C-H), 2212 (C≡C), 1745 (C=O), 1647 (C=O), 1243 (C(O)-O), 1041 (C-O); HRMS (EI/DFS) m/z $[M]^+$ Calcd. for $C_{23}H_{32}O_8N^{32}S_2$: 514.1564; found: 514.1560.

2,2,6a-Triethyl-3-(hydroxymethyl)-1-6-(2-oxoethylidene)hexahydro-1*H*-furo[3,4-*b*]pyrrol-1-oxyl (8)

To a solution of compound **2c** (0.5 g, 2 mmol) in tetrahydrofuran was added manganese(IV) oxide (MnO_2, 5 g, 60 mmol). The reaction mixture was stirred at room temperature for 3 days and monitored by monitored by TLC (SiO_2, ethyl acetate). Upon completion of the reaction, the solid was removed by filtration, and the filtrate was concentrated under reduced pressure. The residue was triturated with ether and the product was collected 0.28 g (50% yield) as yellow crystals, m.p. 100–103 °C. IR (KBr) ν_{max} (cm^{-1}): 3387 (O-H), 2976 (C-H), 2939 (C-H), 2908 (C-H), 2881 (C-H), 2864 (C-H) 1639 (C=O), 1614 (C=C), 1157 (C-O);

Anal. Calcd for $C_{15}H_{24}O_4N$: C, 63.81; H, 8.57; N, 4.96; found: C, 63.68; H, 8.39; N, 4.92; HRMS (EI/DFS) m/z $[M]^+$ Calcd for $C_{15}H_{24}O_4N$: 282.3629; found: 282.3625.

2,2,5-Triethyl-3,4-bis(hydroxymethyl)-5-(3-phenyl-1*H*-pyrazol-5-yl)pyrrolidin-1-oxyl (9a)

The mixture of nitroxide **6a** (200 mg, 0.5 mmol) and hydrazine hydrate (600 mg, 12.0 mmol)in diethyl ether (50 mL) was stirred vigorously at room temperature for 3 days and monitored by monitored by TLC (SiO_2, hexane/EtOAc, 1:1, UV light for visualization). The mixture was washed with 1 M aquous soluton of monosodium phosphate (3 × 15 mL). The organic layer was dried over anhydrous Na_2SO_4, filtered, and concentrated under reduced pressure. The residue was dissolved in methanol (20 mL), and a 2.5 M aqueous NaOH solution (10 mL) was added. After stirring for 2 days, the mixture was diluted with water (50 mL). Methanol was removed in vacuo, and the aqueous layer was extracted with ethyl acetate (3 × 15 mL). The combined organic extracts were dried over Na_2SO_4, filtered, and concentrated under reduced pressure.The residue was triturated with ether and the product 57 mg (33%) was collected as yellow crystals. m.p. 184–186 °C. IR (KBr) ν_{max} (cm^{-1}): 3240 (O-H), 3130 (O-H), 3070 (=C-H), 3053 (=C-H), 3037 (=C-H), 3018 (=C-H), 2966 (C-H), 2939 (C-H), 2881 (C-H); Anal. Calcd. for $C_{21}H_{30}O_3N_3$: C, 67.71; H, 8.12; N, 11.28; found: C, 67.75; H, 8.28; N, 11.26; HRMS (EI/DFS) m/z $[M]^+$ Calcd. for $C_{21}H_{30}O_3N_3$: 372.2282; found: 372.2280; ^1H NMR (300 MHz, CD_3OD, Zn/CF_3COOH, δ): 0.98 (t, J_t=7.3 Hz, 3H), 1.12 (t, J_t=7.5 Hz, 3H), 1.13 (t, J_t=7.3 Hz, 3H); 1.94 (dq, J_d=-15.7 Hz, J_q=7.3 Hz, 1H), 2.00 (dq, J_d=-15.7 Hz, J_q=7.3 Hz, 1H), 2.16 (dq, J_d=-14.3 Hz, J_q=7.5 Hz, 1H), 2.22 (dq, J_d=-9.2 Hz, J_q= 7.3 Hz, 1H), 2.35 (dq, J_d=-14.3 Hz, J_q=7.5 Hz, 1H), 2.57 (dq, J_d=-9.2 Hz, J_q=7.3 Hz, 1H), 2.63; 2.22 (ddd, J_{d1} =6.5 Hz, J_{d2}=7.0 Hz, J_{d3}=11.9 Hz, 1H), 2.63 (ddd, J_{d1}=5.1 Hz, J_{d2}=6.3 Hz, J_{d3}=11.9 Hz, 1H); 3.61 (dd, J_{d1}=7.0 Hz, J_{d2}=-11.4 Hz, 1H), 3.70 (dd, J_{d1}=6.5 Hz, J_{d2}=-11.4 Hz, 1H), 3.73 (dd, J_{d1}=6.3 Hz, J_{d2}=-11.6 Hz, 1H), 3.80 (dd, J_{d1}=5.1 Hz, J_{d2}=-11.6 Hz, 1H); 6.73 (s, 1H); 7.39 (dddd, J_{d1}=1.2 Hz, J_{d2}=1.2 Hz, J_{d3}=7.4 Hz, J_{d4}=7.4 Hz, 1H), 7.47 (dddd, J_{d1}=0.6 Hz, J_{d2}=1.0 Hz, J_{d3}=7.5 Hz, J_{d4}=7.8 Hz, 1H), 7.47 (dddd, J_{d1}=0.6 Hz, J_{d2}=1.0 Hz, J_{d3}=7.4 Hz, J_{d4}=7.8 Hz, 1H), 7.72 (dddd, J_{d1}=0.6 Hz, J_{d2}=1.2 Hz, J_{d3}=1.7 Hz, J_{d4}=7.8 Hz, 1H), 7.72 (dddd, J_{d1}=0.6 Hz, J_{d2}=1.2 Hz, J_{d3}=1.7 Hz, J_d=7.8 Hz, 1H).

Annulated tricyclic pyrazole derivatives synthesis

Method A: A mixture of nitroxide **9a** (2.0 g, 5.4 mmol), *N*,*N*-diisopropylethylamine (2.1 g, 16.0 mmol) and methanesulfonyl chloride (2.3 g, 16.0 mmol) in dry $CHCl_3$ (50 mL) was heated under reflux for 30 min. The reaction was monitored by TLC (SiO_2, hexane:diethyl ether

1:1, UV light for visualization). The mixture was washed with hot water (60 °C, 3 × 50 mL). The organic phase was evaporated under reduced pressure, and the crude residue was purified by column chromatography (SiO_2 hexane:diethyl ether = 1:4) to afford the two products **9b** (1.0 g, 43%) and **9c** (0.5 g, 26%).

Method B: A mixture of nitroxide **9a** (2.0 g, 5.4 mmol), *N*,*N*-diisopropylethylamine (4.2 g, 32.0 mmol) and methanesulfonyl chloride (3.9 g, 27.0 mmol) in dry $CHCl_3$ (50 mL) was heated under reflux for 30 min. The reaction was monitored by TLC (SiO_2, hexane:diethyl ether 1:1, UV light for visualization). The mixture was washed with hot water (60 °C, 3 × 50 mL). The organic phase was evaporated under reduced pressure, and the crude residue was purified by column chromatography (SiO_2 hexane:diethyl ether = 1:4) to afford 1.5 g (65%) of nitroxide **9b**.

Method C.

The mixture nitroxide **6b** (200 mg, 0.4 mmol) and hydrazine hydrate (250 mg, 5.0 mmol) in diethyl ether (10 mL) was stirred vigorously at room temperature for 1 day and monitored by TLC (SiO_2, hexane/ether, 1:4, UV light for visualization). The mixture was washed with 1 M aquous soluton of monosodium phosphate (3 × 5 mL). The organic layer was dried over anhydrous Na_2SO_4, filtered, and concentrated under reduced pressure. The residue was dissolved in methanol (20 mL) and stirred under an ambient atmosphere for 24 hours to oxidation. The solvent was evaporated under reduced pressure, and the crude residue was purified by column chromatography (SiO_2, hexane:diethyl ether = 1:4) to afford the product as an oily substance **9b** (73 mg, 42%).

3b,5,5-Triethyl-6-(((methylsulfonyl)oxy)methyl)-2-phenyl-3b,4,5,6,6a,7-hexahydropyrrolo[2',3':3,4]pyrrolo[1,2-*b*]pyrazol-4-oxyl (9b)

Yellow oil. IR (neat) ν_{max} (cm^{-1}): 3135 (=C-H), 3018 (=C-H), 2976 (C-H), 2939 (C-H), 2883 (C-H), 1352 (S=O), 1176 (S=O); HRMS (EI/DFS) m/z $[M]^+$ Calcd for $C_{22}H_{30}O_4N_3{}^{32}S$: 432.1952; found: 432.1953; ^1H NMR (300 MHz, CD_3OD, Zn/CF_3COOH, δ) 0.91 (t, J_t=7.3 Hz, 3H), 0.95 (t, J_t=7.3 Hz, 3H), 1.09 (t, J_t=7.3 Hz, 3H); 1.23 (dq, J_d=-14.5 Hz, J_q=7.3 Hz, 1H), 1.68 (dq, J_d=-14.5 Hz, J_q=7.3 Hz, 1H), 1.98 (dq, J_d=-15.3 Hz, J_q=7.3 Hz, 1H), 2.03 (dq, J_d=-15.3 Hz, J_q=7.3 Hz, 1H), 2.20 (dq, J_d=-13.7, Hz, J_q=7.3 Hz, 1H), 2.36 (dq, J_d=-13.7, Hz, J_q=7.3 Hz, 1H); 2.81 (ddd, J_d=5.8 Hz, J_d=5.3 Hz, J_d=4.9 Hz, 1H), 3.65 (ddd, J_d=7.7 Hz, J_d=5.8 Hz, J_d=1.9 Hz, 1H); 3.23 (s, 3H); 4.40 (dd, J_{d1}=-11.9 Hz, J_{d2}=1.9 Hz, 1H), 4.51 (dd, J_{d1} =-10.8 Hz, J_{d2}=4.9 Hz, 1H), 4.58 (dd, J_{d1}=-10.8 Hz, J_{d2}=5.3 Hz, 1H), 4.60 (dd, J_{d1}=-11.9 Hz, J_{d2}=7.7 Hz, 1H); 6.80 (s, 1H); 7.35 (dddd, J_{d1}=7.1=4 Hz, J_{d2}=7.4 Hz, J_{d3}=1.1 Hz, J_{d4}=1.1 Hz, 1H), 7.42 (dddd, J_{d1}=7.8 Hz, J_{d2}=7.4 Hz, J_{d3}=1.1 Hz, J_{d4}=0.6 Hz, 1H), 7.42 (dddd, J_{d1}=7.8 Hz, J_{d2}=7.4 Hz, J_{d3}=1.1 Hz, J_{d4}=0.7

Hz, 1H), 7.80 (dddd, J_{d1}=7.8 Hz, J_{d2}=1.7 Hz, J_{d3}=1.1 Hz, J_{d4}=0.6 Hz, 1H), 7.80 (dddd, J_{d1}=7.8 Hz, J_{d2}=1.7 Hz, J_{d3}=1.1 Hz, J_{d4}=0.6 Hz, 1H).

3b,5,5-Triethyl-6-(hydroxymethyl)-2-phenyl-3b,4,5,6,6a,7-hexahydropyrrolo[2',3':3,4]pyrrolo[1,2-*b*]pyrazol-4-oxyl (9c)

Yellow crystals, m.p. 56-59 °C. IR (KBr) ν_{max} (cm^{-1}): 3356 (O-H), 3062 (=C-H), 3037 (=C-H), 2970 (C-H), 2937 (C-H), 2881 (C-H); HRMS (EI/DFS) m/z $[M]^+$ Calcd for $C_{21}H_{28}O_2N_3$: 354.2176; found: 354.2171; ^{1}H NMR (300 MHz, CD_3OD, Zn/CF_3COOH, δ) 0.87 (t, J_t=7.3 Hz, 3H), 0.94 (t, J_t=7.3 Hz, 3H), 1.05 (t, J_t=7.3 Hz, 3H); 1.18 (dq, J_d=-14.4 Hz, J_q=7.3 Hz, 1H), 1.49 (dq, J_d=-14.4 Hz, J_q=7.3 Hz, 1H), 2.00 (dq, J_d=-15.1 Hz, J_q=7.3 Hz, 1H), 2.10 (dq, J_d=-15.1 Hz, J_q=7.3 Hz, 1H), 2.27 (dq, J_d=-13.5, Hz, J_q=7.3 Hz, 1H), 2.34 (dq, J_d=-13.5, Hz, J_q=7.3 Hz, 1H); 2.49 (ddd, J_d=3.5 Hz, J_d=3.5 Hz, J_d=3.7 Hz, 1H), 3.65 (ddd, J_d=2.5 Hz, J_d=3.7 Hz, J_d=8.8 Hz, 1H); 3.81 (dd, J_{d1}=-10.8 Hz, J_{d2}=3.5 Hz, 1H), 3.88 (dd, J_{d1} =-10.8 Hz, J_{d2}=3.5 Hz, 1H), 4.33 (dd, J_{d1}=-12.1 Hz, J_{d2}=2.5 Hz, 1H), 4.62 (dd, J_{d1}=-12.1 Hz, J_{d2}=8.7 Hz, 1H); 6.75 (s, 1H); 7.34 (dddd, J_{d1}=7.1=4 Hz, J_{d2}=7.4 Hz, J_{d3}=1.2 Hz, J_{d4}=1.2 Hz, 1H), 7.42 (dddd, J_{d1}=7.8 Hz, J_{d2}=7.4 Hz, J_{d3}=1.0 Hz, J_{d4}=0.6 Hz, 1H), 7.42 (dddd, J_{d1}=7.8 Hz, J_{d2}=7.4 Hz, J_{d3}=1.0 Hz, J_{d4}=0.6 Hz, 1H), 7.80 (dddd, J_{d1}=7.8 Hz, J_{d2}=1.8 Hz, J_{d3}=1.2 Hz, J_{d4}=0.6 Hz, 1H), 7.80 (dddd, J_{d1}=7.8 Hz, J_{d2}=1.8 Hz, J_{d3}=1.2 Hz, J_{d4}=0.6 Hz, 1H).

NMR spectra

Figure S1. ^{1}H NMR (300 MHz, CD_3OD, Zn/CF_3COOH) of 2,2,5-triethyl-3,4-bis(hydroxymethyl)-5-((trimethylsilyl)ethynyl)pyrrolidin-1-oxyl (2d)

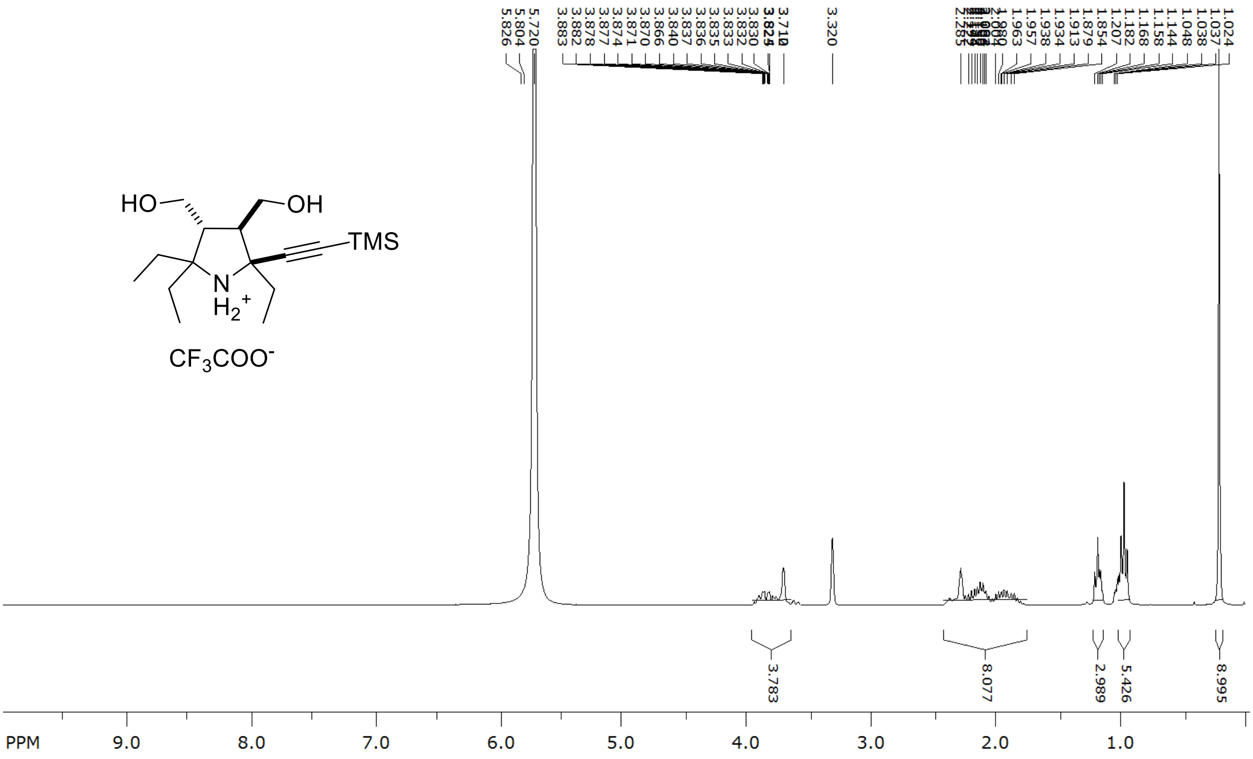

Figure S2. ^{1}H NMR (300 MHz, CD_3OD, Zn/CF_3COOH) of 2-(3-(benzyloxy)prop-1-yn-1-yl)-2,5,5-triethyl-3,4-bis(hydroxymethyl)pyrrolidin-1-oxyl (2e)

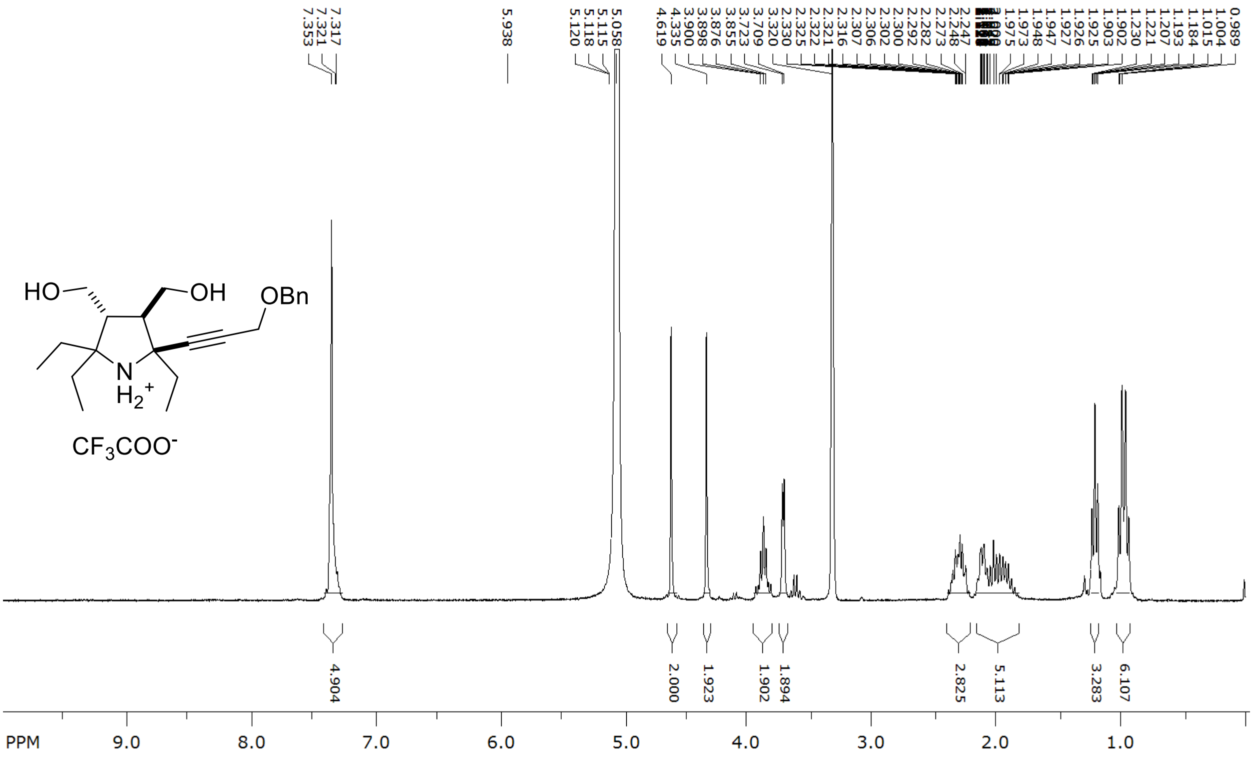

Figure S3. ^{1}H **NMR (500 MHz, CD_3OD, Zn/CF_3COOH) of 2-(3-(dimethylamino)prop-1-yn-1-yl)-2,5,5-triethyl-3,4-bis(hydroxymethyl)pyrrolidin-1-oxyl (2f)**

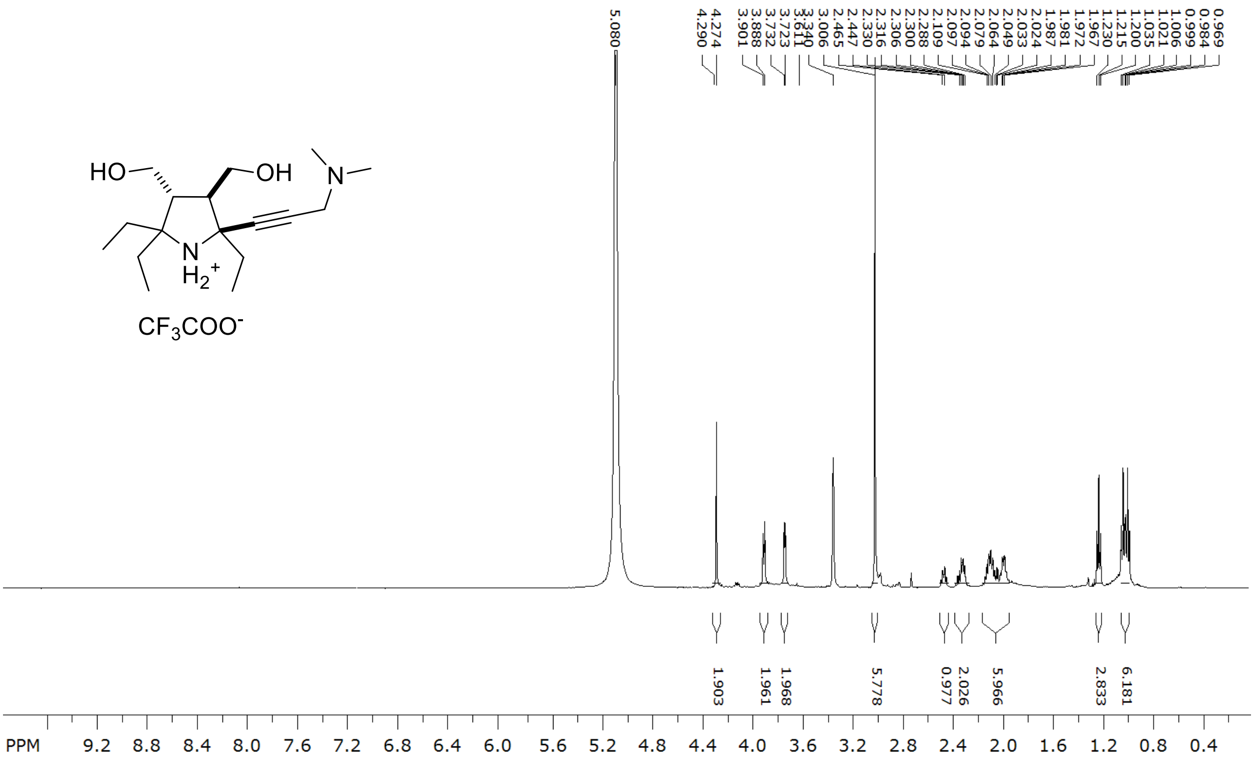

Figure S4. ^{1}H **NMR (300 MHz, CD_3OD, Zn/CF_3COOH) of 2,2,5-triethyl-5-ethynyl-3,4-bis(((methylsulfonyl)oxy)methyl)pyrrolidin-1-oxyl (3a)**

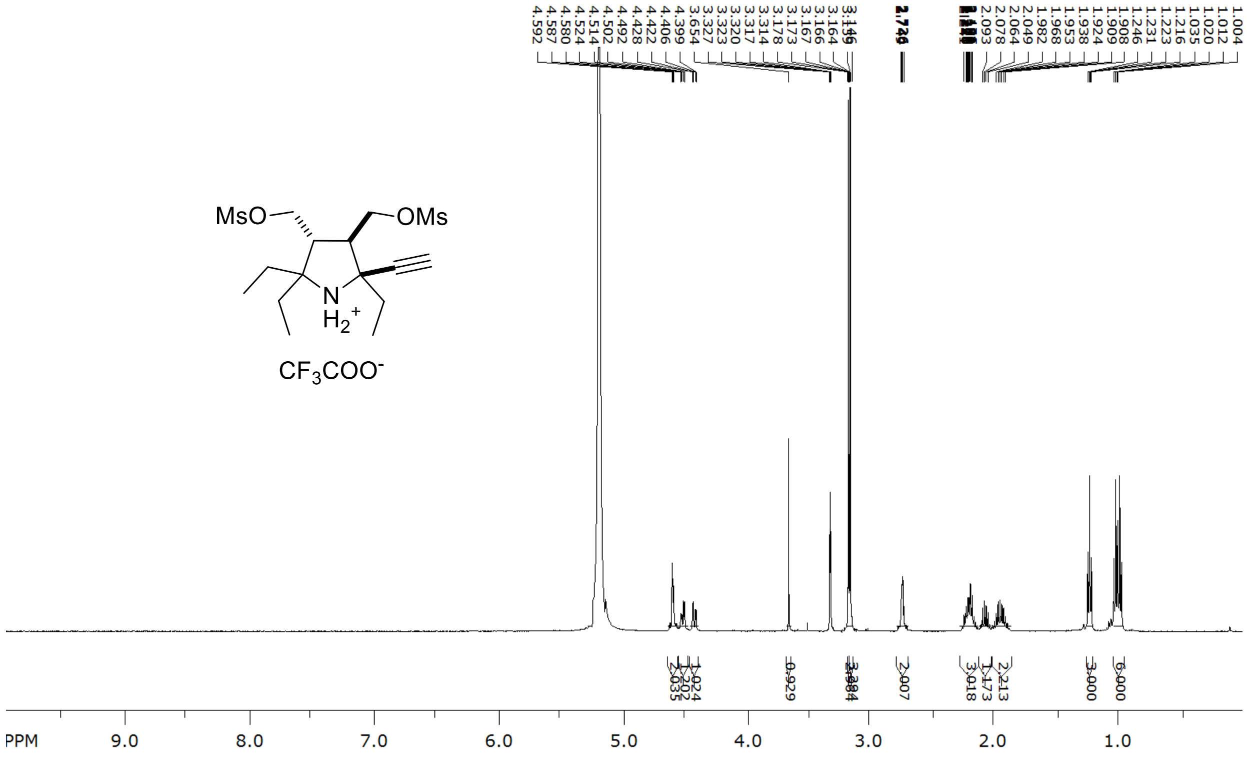

Figure S5. ^{1}H NMR (300 MHz, CD_3OD, Zn/CF_3COOH) of 2,2,5-triethyl-3,4-bis(((methylsulfonyl)oxy)methyl)-5-(phenylethynyl)pyrrolidin-1-oxyl (3b)

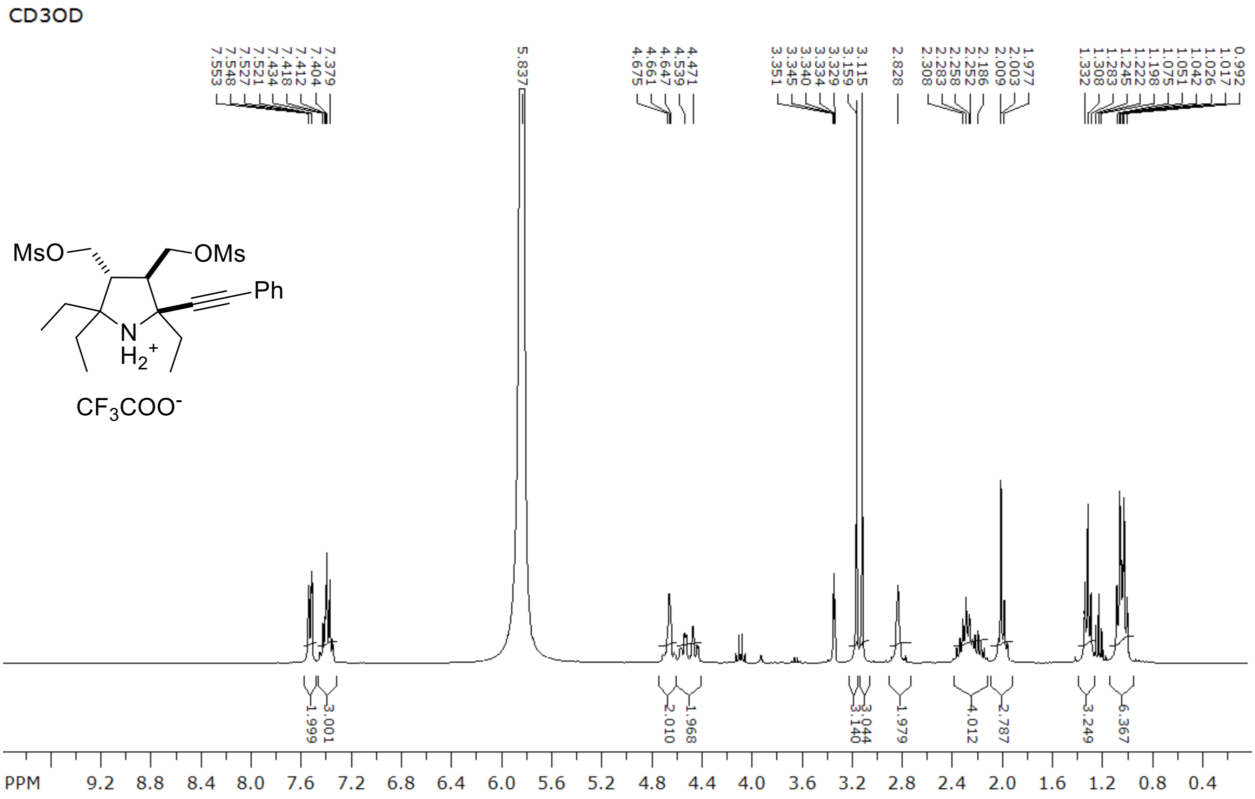

Figure S6. ^{1}H NMR (300 MHz, CD_3OD, Zn/CF_3COOH) of 2,2,5-triethyl-3,4-bis(((methylsulfonyl)oxy)methyl)-5-((trimethylsilyl)ethynyl)pyrrolidin-1-oxyl (3d)

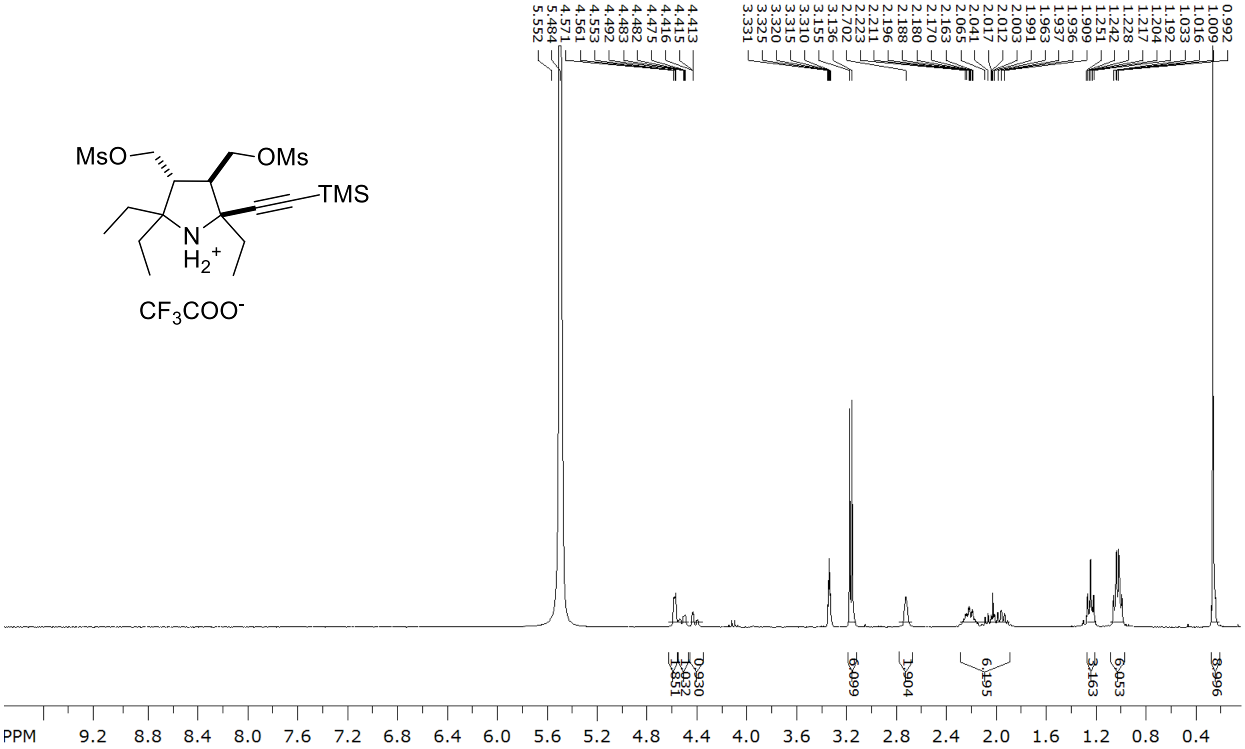

Figure S7. **^{1}H NMR (300 MHz, CD_3OD, Zn/CF_3COOH) of 2-(3-(dimethylamino)prop-1-yn-1-yl)-2,5,5-triethyl-3,4-bis(((methylsulfonyl)oxy)methyl)pyrrolidin-1-oxyl (3e)**

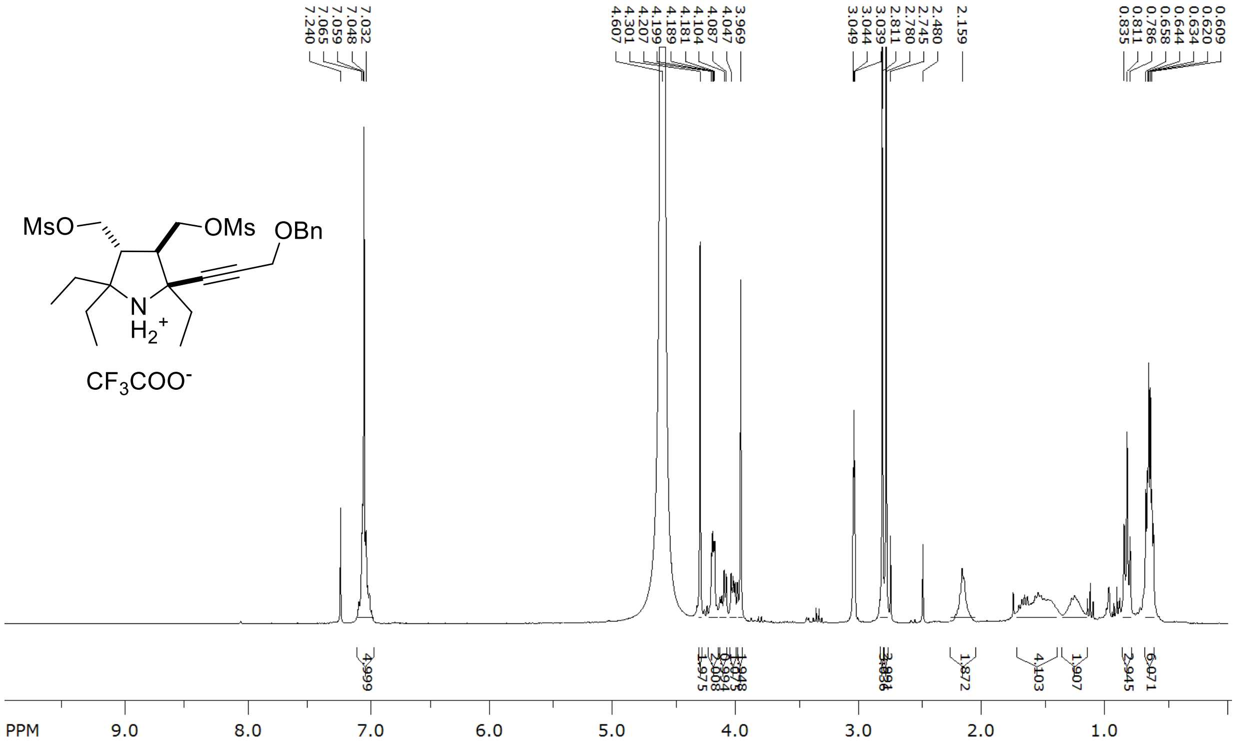

Figure S8. **^{1}H NMR (400 MHz, CD_3OD, Zn/CF_3COOH) of 2-(3-(dimethylamino)prop-1-yn-1-yl)-2,5,5-triethyl-3,4-bis(((methylsulfonyl)oxy)methyl)pyrrolidin-1-oxyl (3f)**

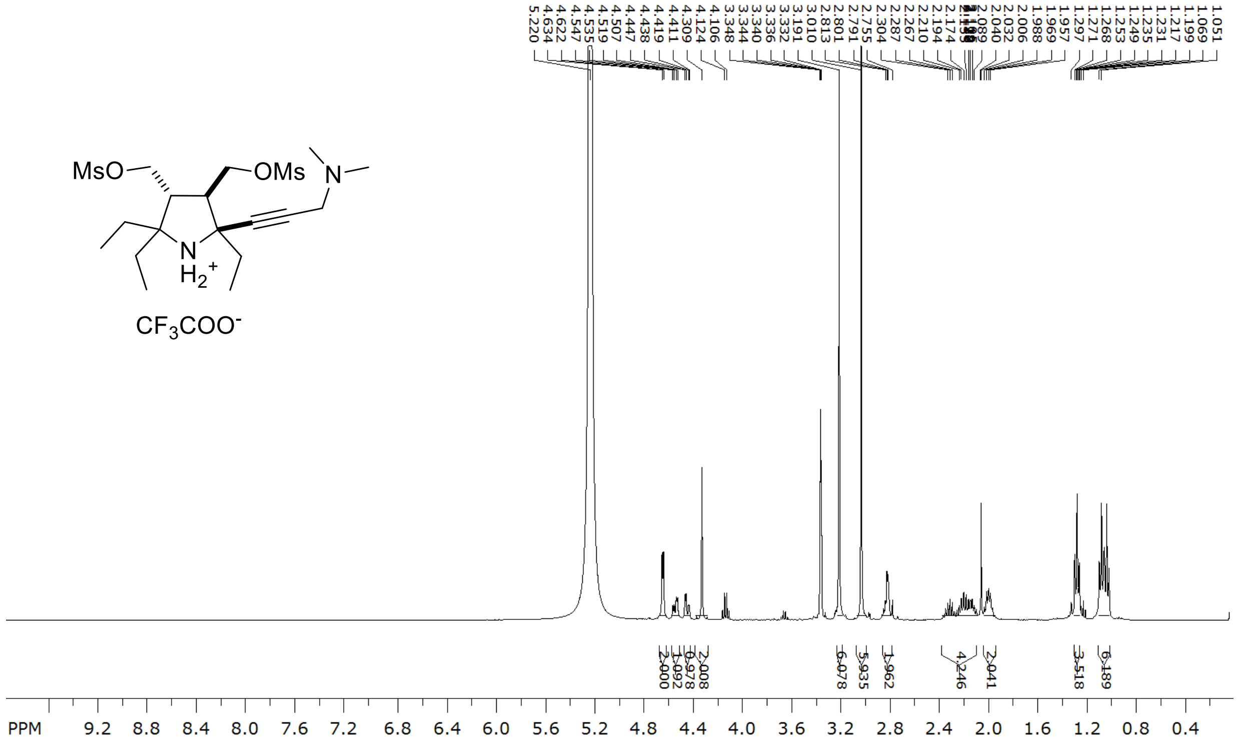

Figure S9. **^{1}H NMR (400 MHz, CD_3OD, Zn/CF_3COOH) of 3,4-bis(acetoxymethyl)-2,2,5-triethyl-5-ethynylpyrrolidin-1-oxyl (5)**

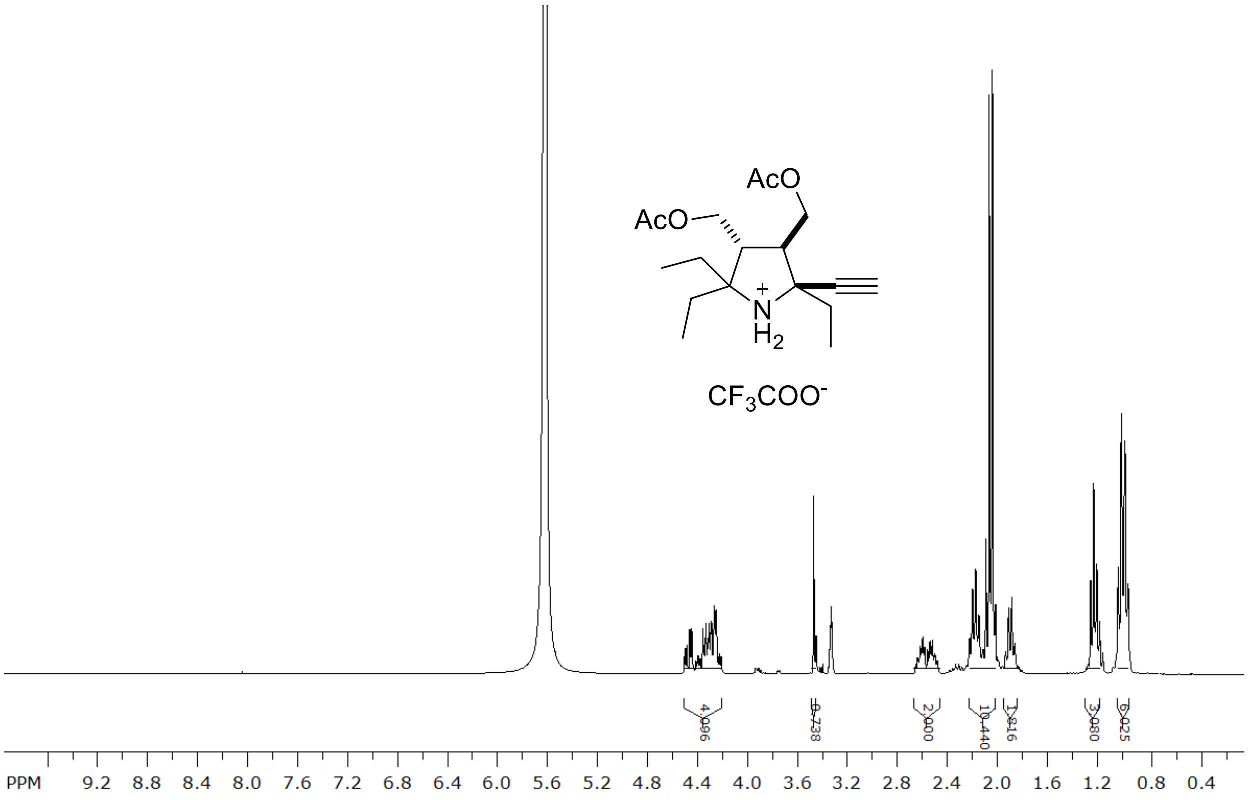

Figure S10. **^{1}H NMR (300 MHz, CD_3OD, Zn/CF_3COOH) of 2,2,5-triethyl-3,4-bis(hydroxymethyl)-5-(3-phenyl-1*H*-pyrazol-5-yl)pyrrolidin-1-oxyl (9a)**

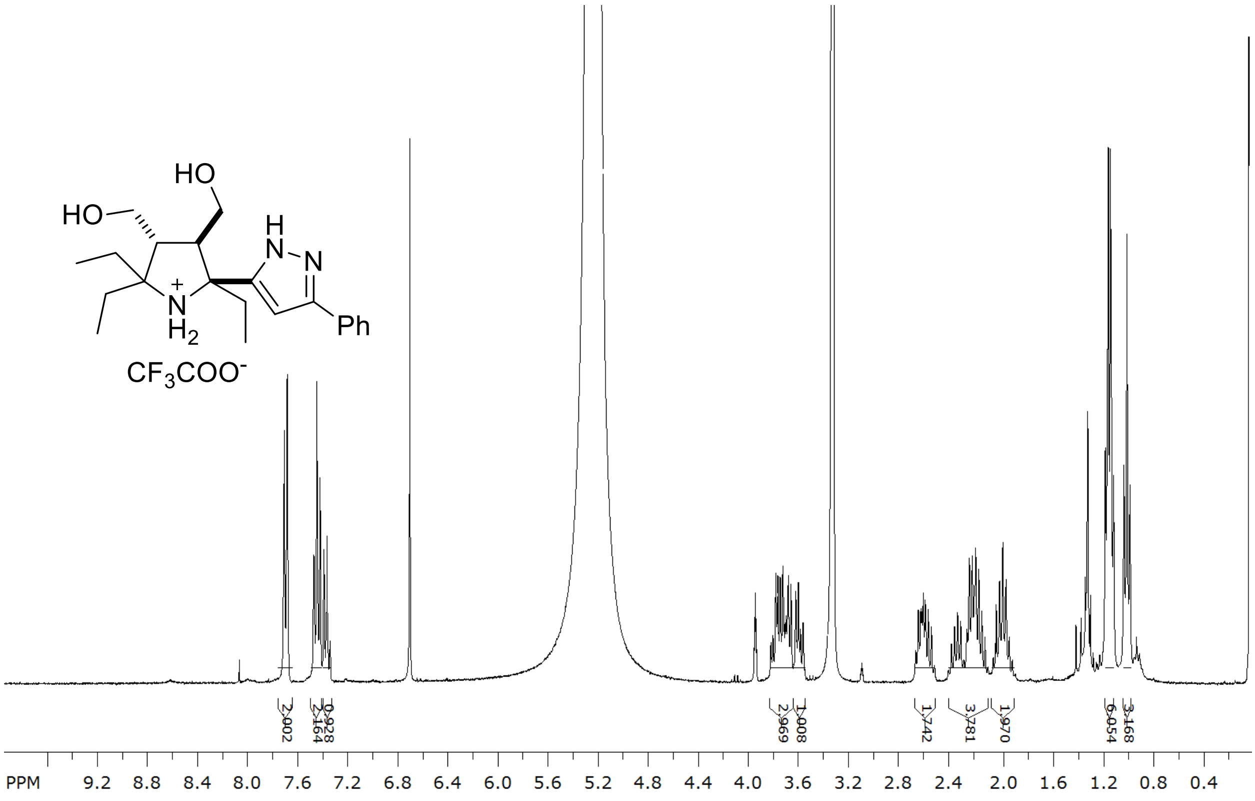

Figure S11. **^{1}H NMR (300 MHz, CD_3OD, Zn/CF_3COOH) of 3b,5,5-triethyl-6-(((methylsulfonyl)oxy)methyl)-2-phenyl-3b,4,5,6,6a,7-hexahydropyrrolo[2',3':3,4]pyrrolo[1,2-*b*]pyrazol-4-oxyl (9b)**

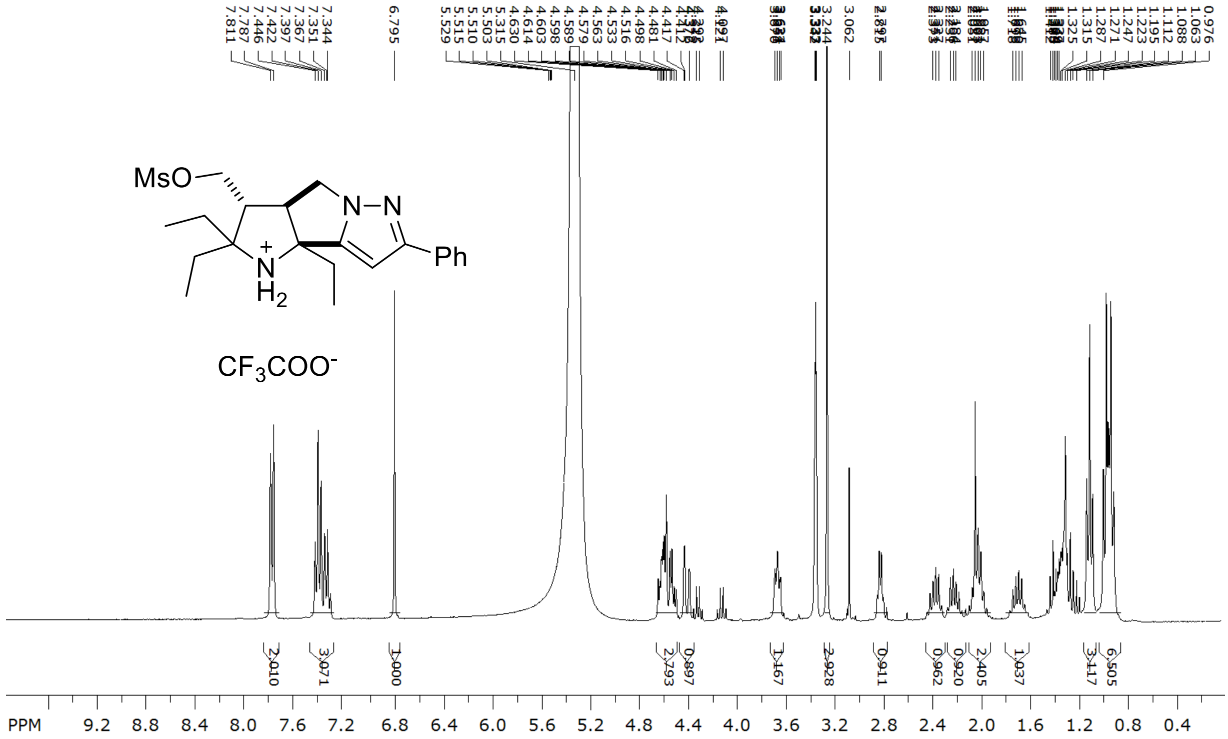

Figure S12. **^{1}H NMR (300 MHz, CD_3OD, Zn/CF_3COOH) of 3b,5,5-triethyl-6-(hydroxymethyl)-2-phenyl-3b,4,5,6,6a,7-hexahydropyrrolo[2',3':3,4]pyrrolo[1,2-*b*]pyrazol-4-oxyl (9c)**

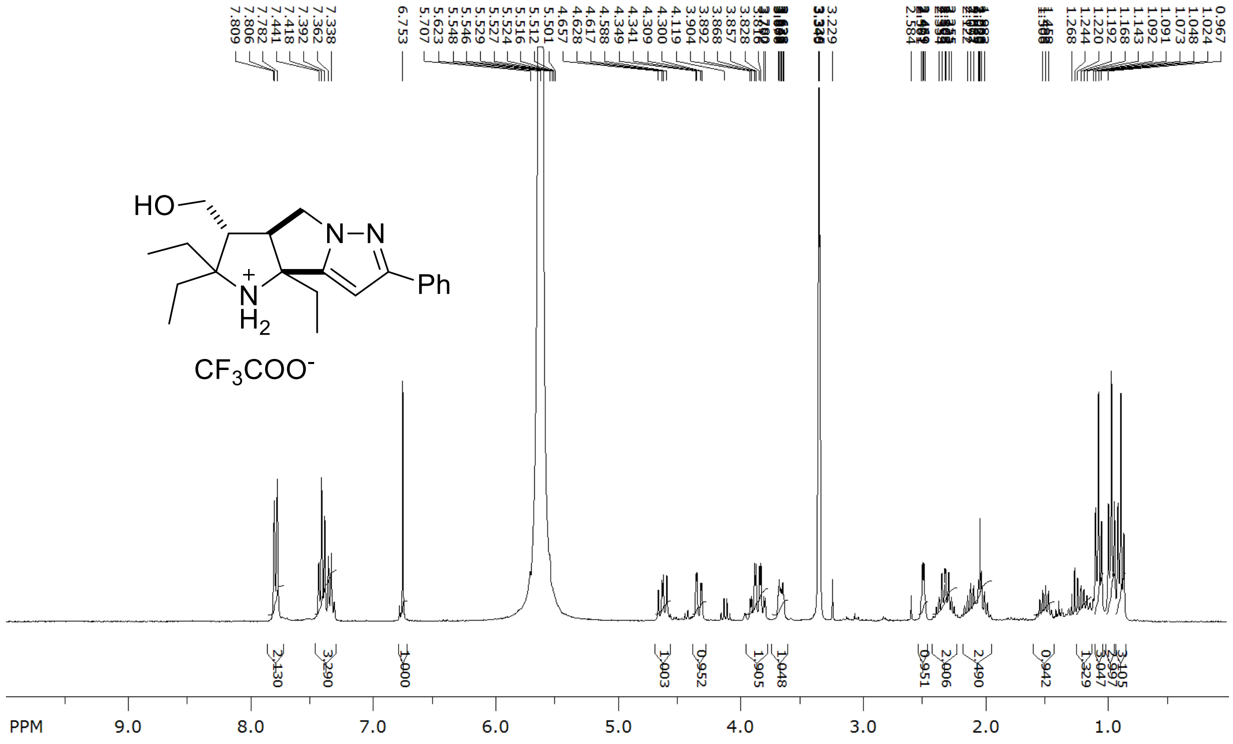

IR spectra

Figure S13. IR (KBr) of 2,2,5-triethyl-3,4-bis(hydroxymethyl)-5-((trimethylsilyl)ethynyl)pyrrolidin-1-oxyl (2d)

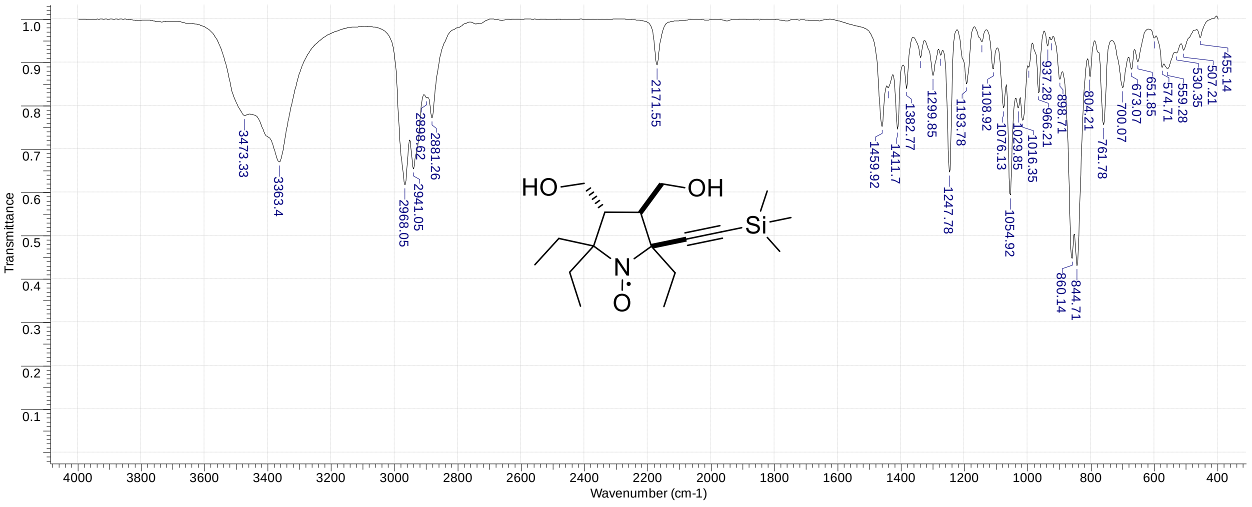

Figure S14. IR (neat) of 2-(3-(benzyloxy)prop-1-yn-1-yl)-2,5,5-triethyl-3,4-bis(hydroxymethyl)pyrrolidin-1-oxyl (2e)

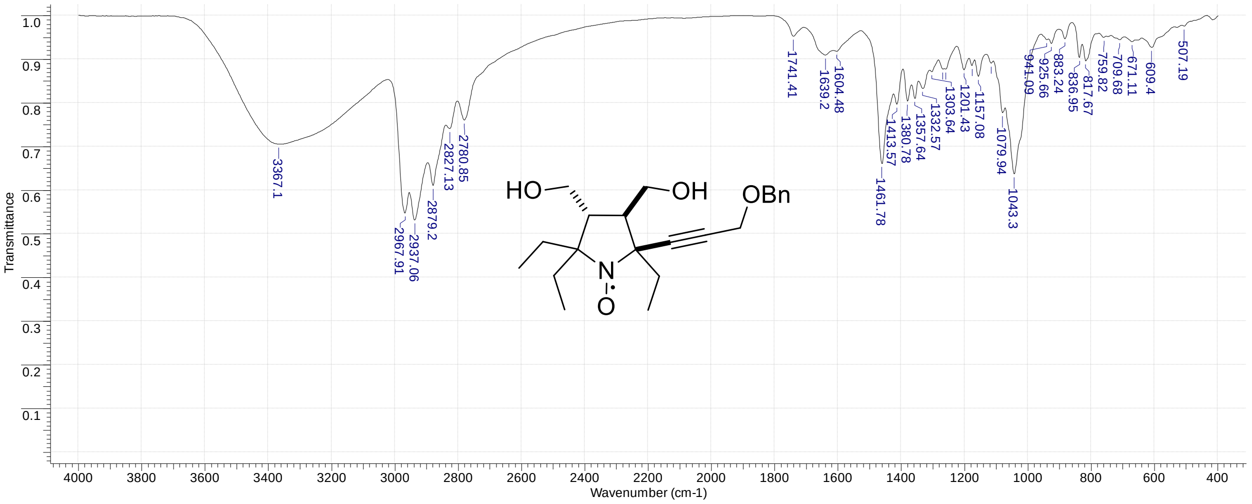

Figure S15. IR (neat) of 2-(3-(dimethylamino)prop-1-yn-1-yl)-2,5,5-triethyl-3,4-bis(hydroxymethyl)pyrrolidin-1-oxyl (2f)

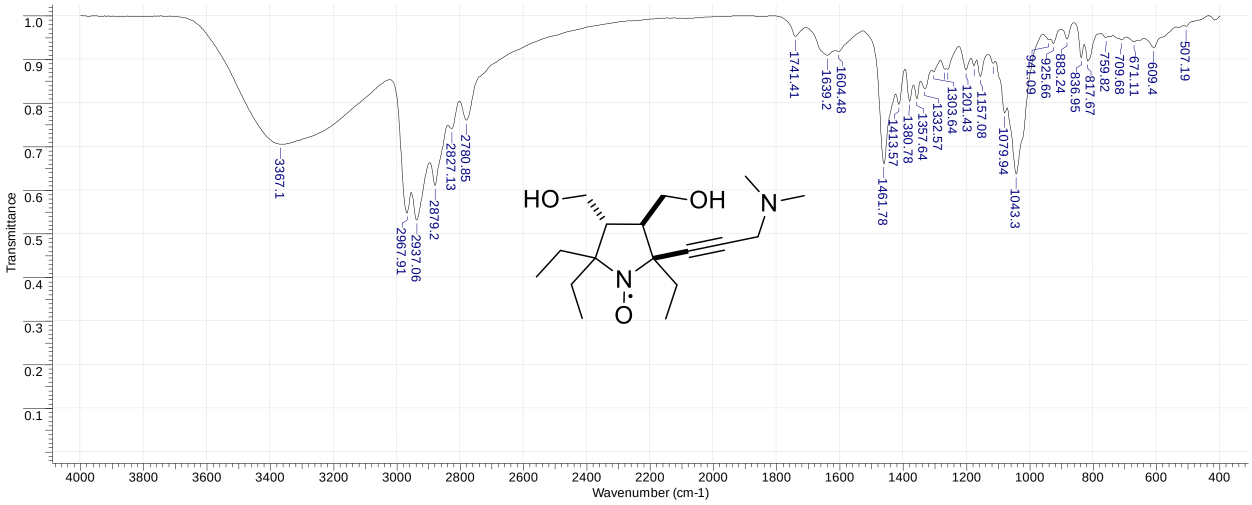

Figure S16. IR (KBr) of 2,2,5-triethyl-5-ethynyl-3,4-bis(((methylsulfonyl)oxy)methyl)pyrrolidin-1-oxyl (3a)

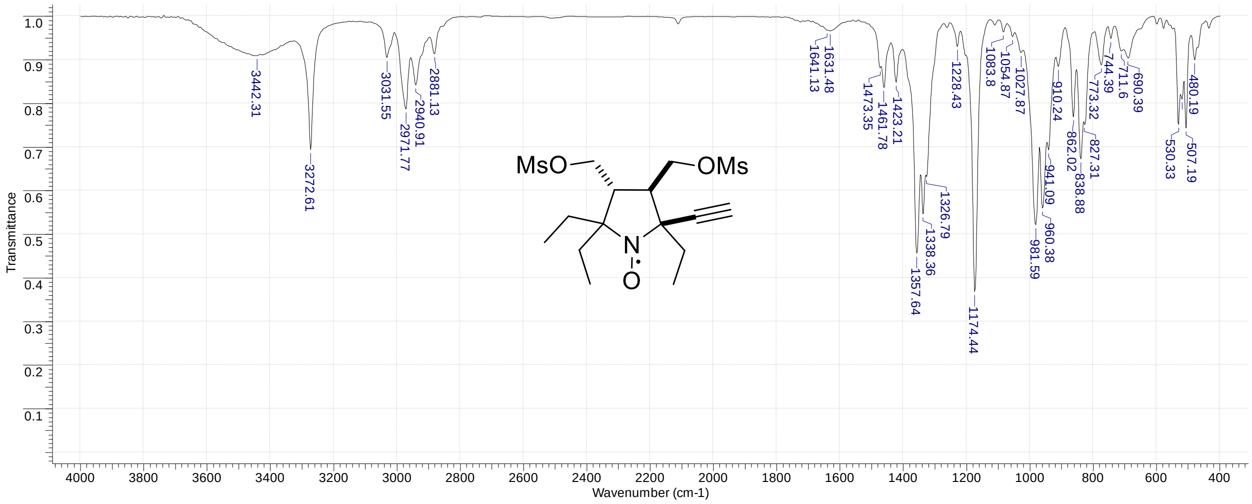

Figure S17. IR (neat) of 2,2,5-triethyl-3,4-bis(((methylsulfonyl)oxy)methyl)-5-(phenylethynyl)pyrrolidin-1-oxyl (3b)

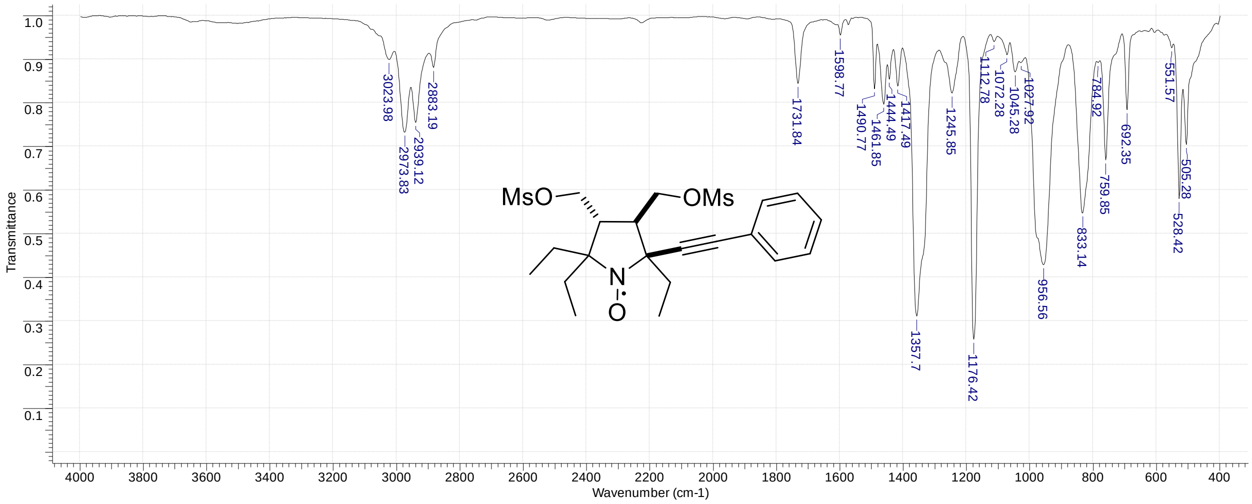

Figure S18. **IR (neat) of 2,2,5-triethyl-3,4-bis(((methylsulfonyl)oxy)methyl)-5-(3-((methylsulfonyl)oxy)prop-1-yn-1-yl)pyrrolidin-1-oxyl (3c)**

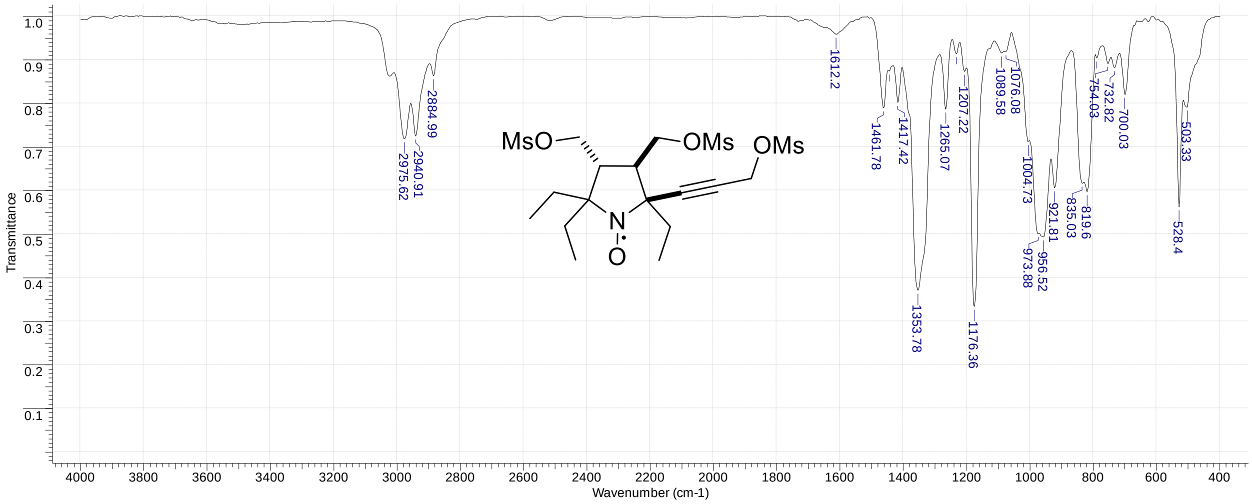

Figure S19. **IR (neat) of 2,2,5-triethyl-3,4-bis(((methylsulfonyl)oxy)methyl)-5-((trimethylsilyl)ethynyl)pyrrolidin-1-oxyl (3d)**

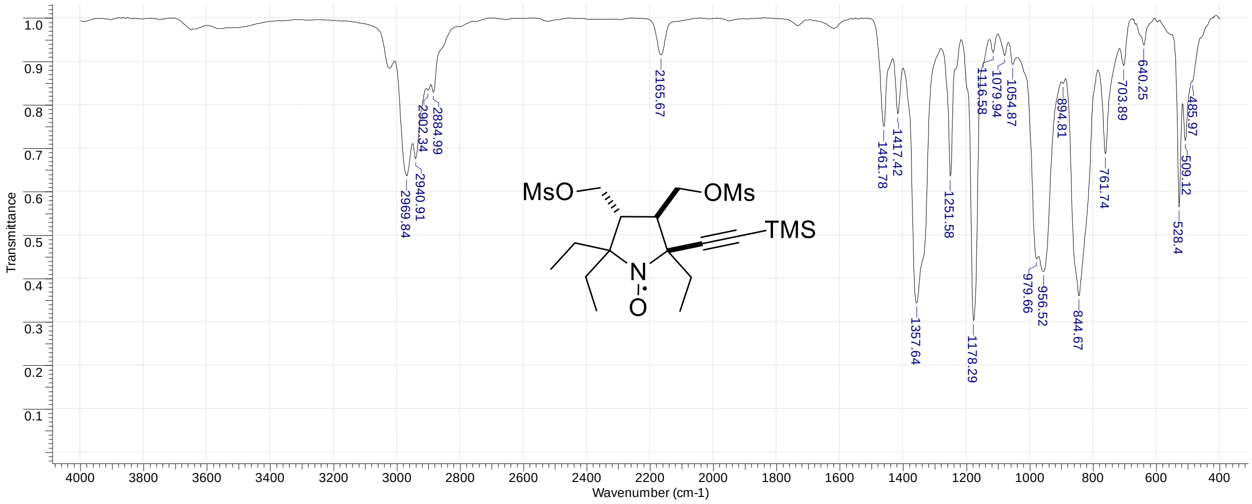

Figure S20. **IR (neat) of 2-(3-(benzyloxy)prop-1-yn-1-yl)-2,5,5-triethyl-3,4-bis(((methylsulfonyl)oxy)methyl)pyrrolidin-1-oxyl (3e)**

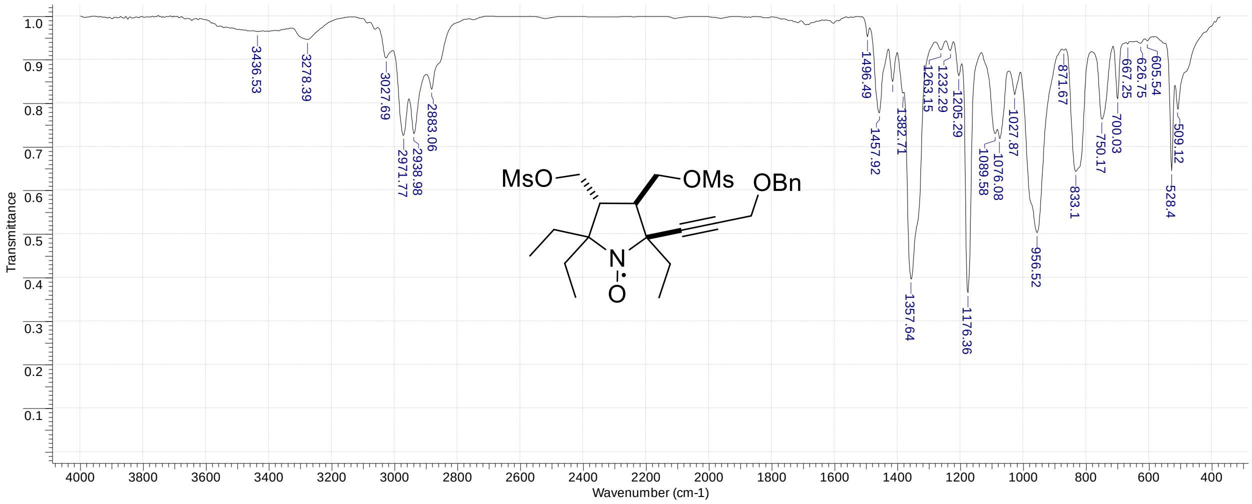

Figure S21. IR (neat) of 2-(3-(dimethylamino)prop-1-yn-1-yl)-2,5,5-triethyl-3,4-bis(((methylsulfonyl)oxy)methyl)pyrrolidin-1-oxyl (3f)

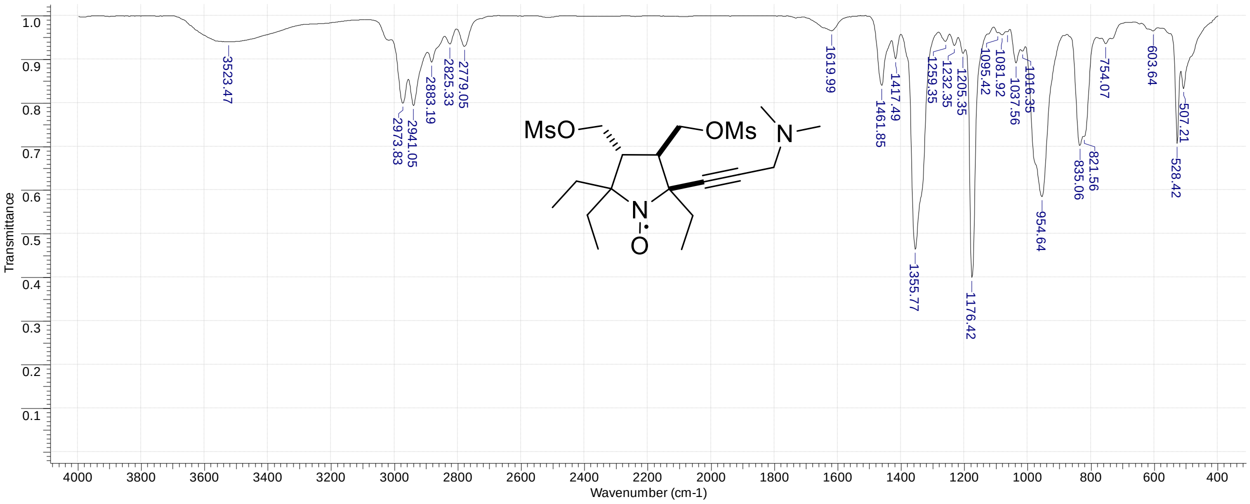

Figure S22. IR (KBr) of 6-(azidomethyl)-3b,5,5-triethyl-3b,4,5,6,6a,7-hexahydropyrrolo[2',3':3,4]pyrrolo[1,2-*c*][1,2,3]triazol-4-oxyl (4a)

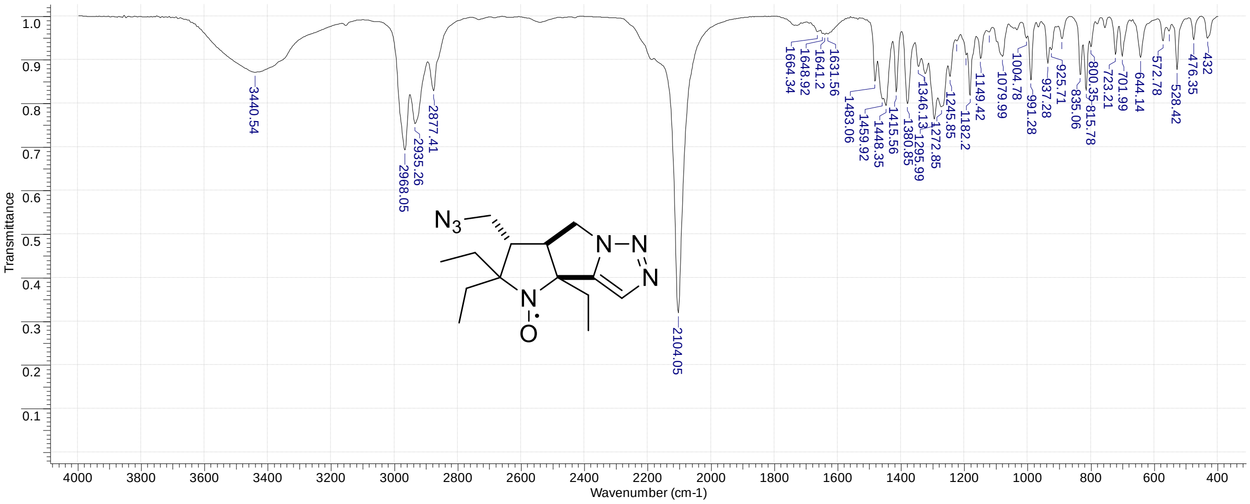

Figure S23. IR (KBr) of 6-(azidomethyl)-3b,5,5-triethyl-3-phenyl-3b,4,5,6,6a,7-hexahydropyrrolo[2',3':3,4]pyrrolo[1,2-*c*][1,2,3]triazol-4-oxyl (4b)

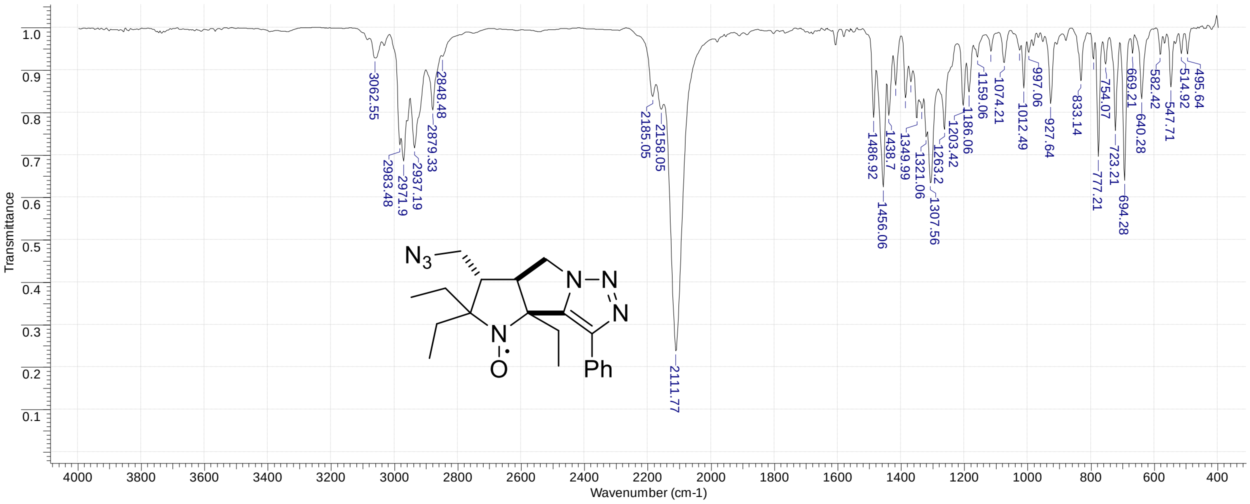

Figure S24. IR (KBr) of 3,6-bis(azidomethyl)-3b,5,5-triethyl-3b,4,5,6,6a,7-hexahydropyrrolo[2',3':3,4]pyrrolo[1,2-*c*][1,2,3]triazol-4-oxyl (4c)

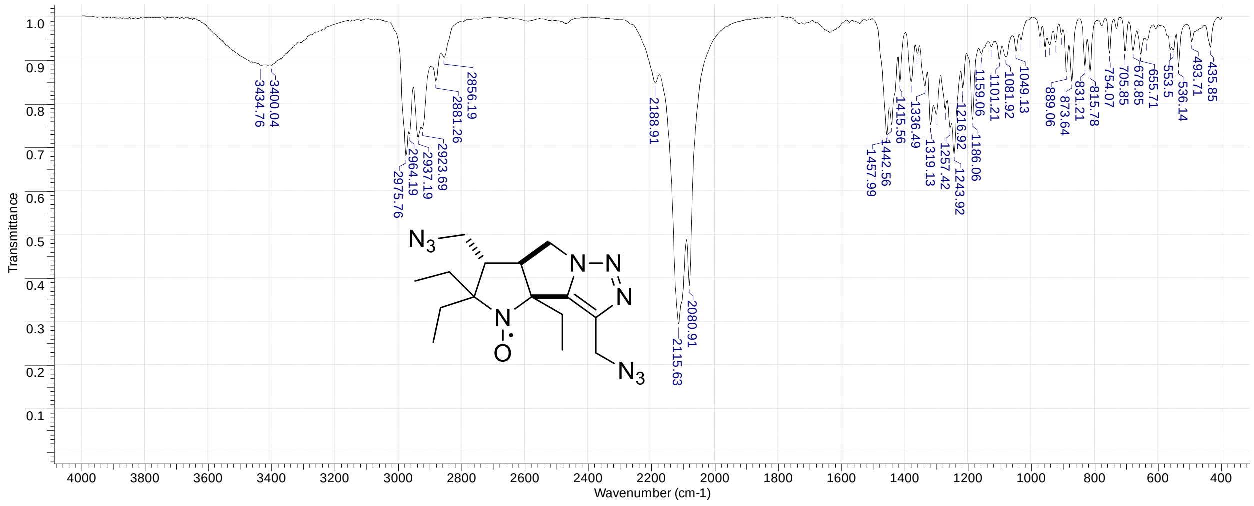

Figure S25. IR (KBr) of 6-(azidomethyl)-3b,5,5-triethyl-3-(trimethylsilyl)-3b,4,5,6,6a,7-hexahydropyrrolo[2',3':3,4]pyrrolo[1,2-*c*][1,2,3]triazol-4-oxyl (4d)

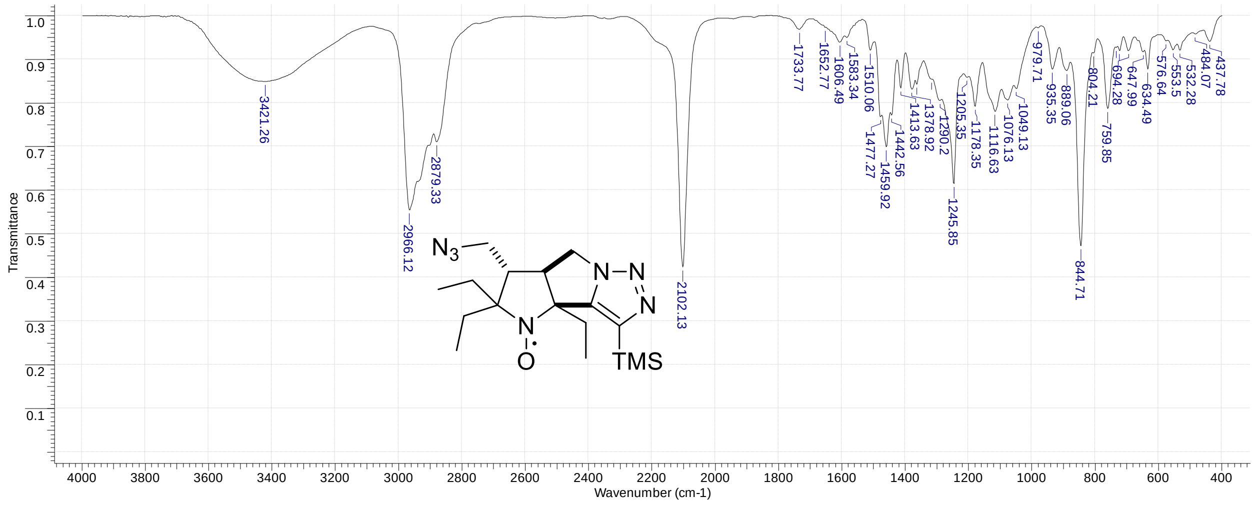

Figure S26. IR (neat) of 6-(azidomethyl)-3-((benzyloxy)methyl)-3b,5,5-triethyl-3b,4,5,6,6a,7-hexahydropyrrolo[2',3':3,4]pyrrolo[1,2-*c*][1,2,3]triazol-4-oxyl (4e)

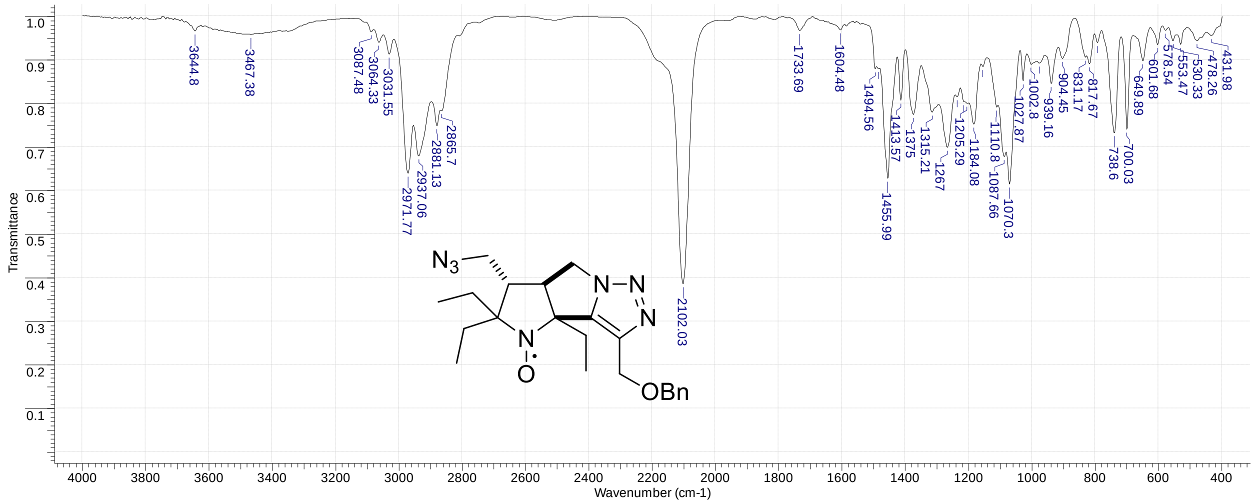

Figure S27. IR (neat) of 6-(azidomethyl)-3-((dimethylamino)methyl)-3b,5,5-triethyl-3b,4,5,6,6a,7-hexahydropyrrolo[2',3':3,4]pyrrolo[1,2-*c*][1,2,3]triazol-4-oxyl (4f)

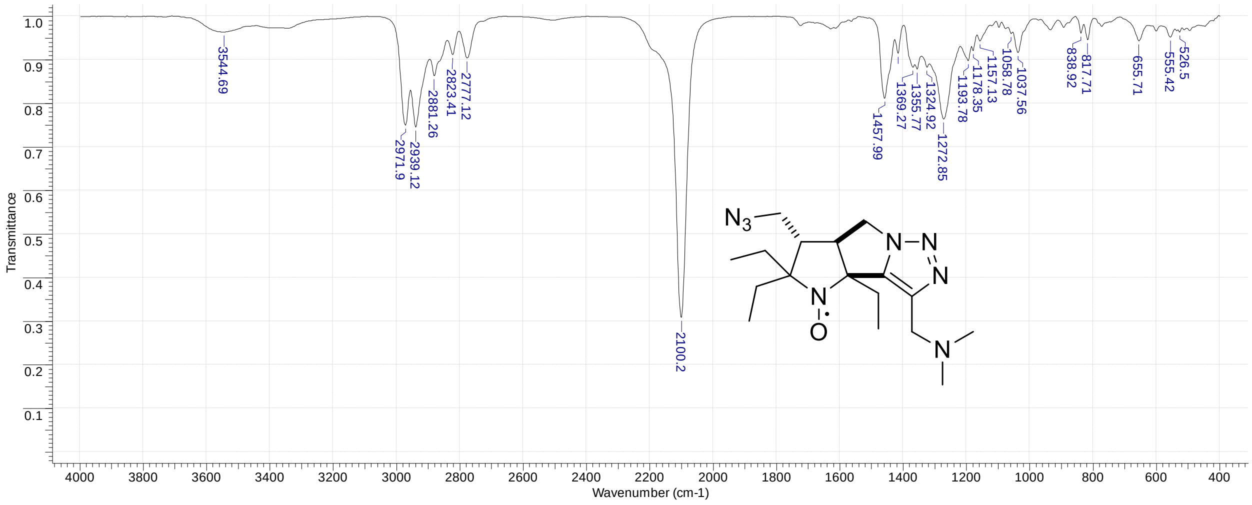

Figure S28. IR (neat) of 3,4-bis(acetoxymethyl)-2,2,5-triethyl-5-ethynylpyrrolidin-1-oxyl (5)

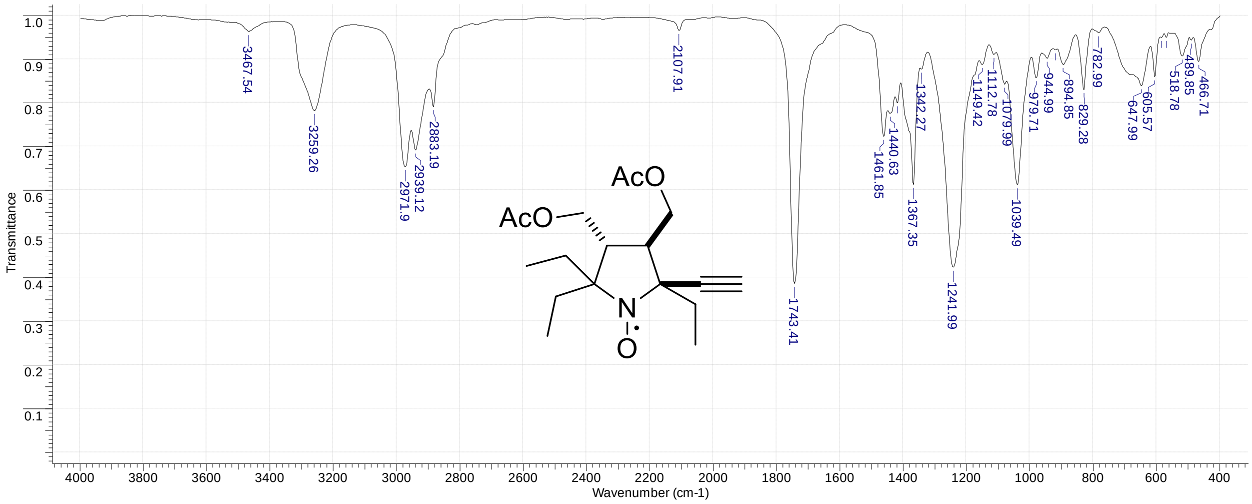

Figure S29. IR (neat) of 3,4-bis(acetoxymethyl)-2,2,5-triethyl-5-(3-oxo-3-phenylprop-1-yn-1-yl)pyrrolidin-1-oxyl (6a)

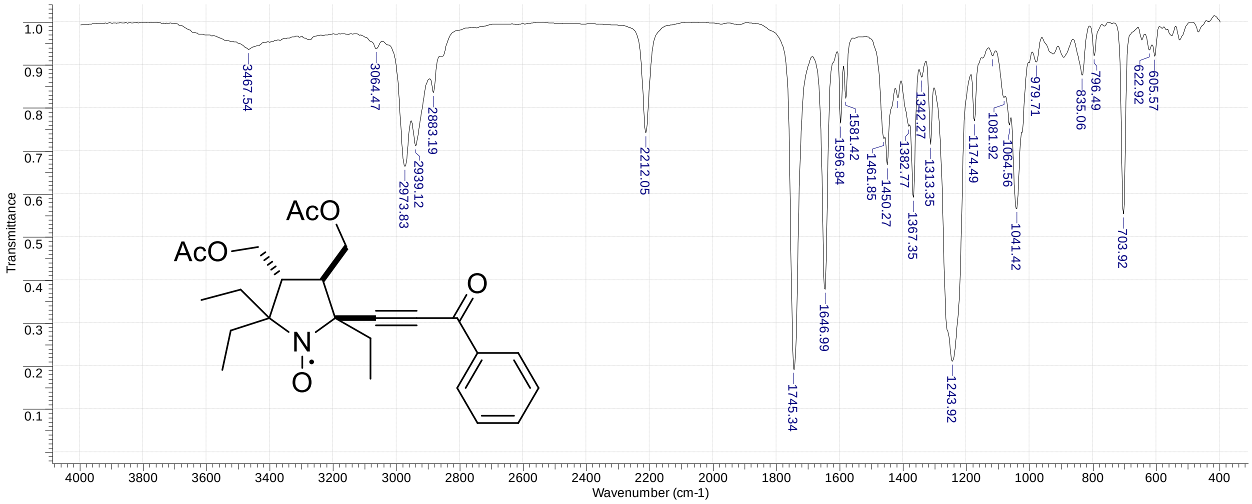

Figure S30. IR (neat) of 2,2,5-triethyl-3,4-bis(((methylsulfonyl)oxy)methyl)-5-(3-oxo-3-phenylprop-1-yn-1-yl)pyrrolidin-1- oxyl (6b)

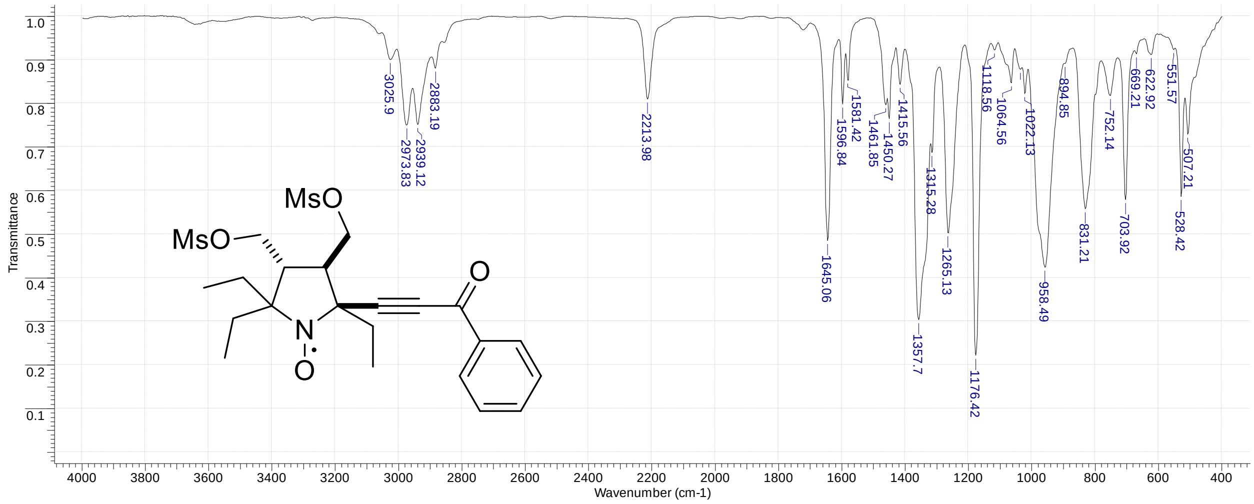

Figure S31. IR (KBr) of (Z)-2,2,6a-triethyl-3-(hydroxymethyl)-6-(2-oxoethylidene)hexahydro-1*H*-furo[3,4-*b*]pyrrol-1-oxyl (8)

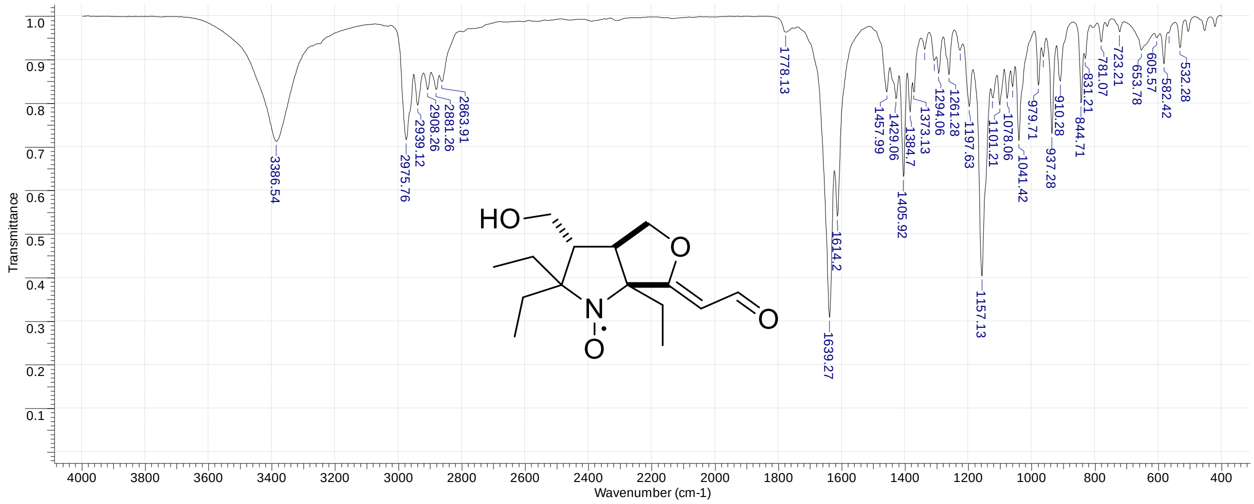

Figure S32. IR (neat) 2,2,5-triethyl-3,4-bis(hydroxymethyl)-5-(3-phenyl-1*H*-pyrazol-5-yl)pyrrolidin-1-oxyl (9a)

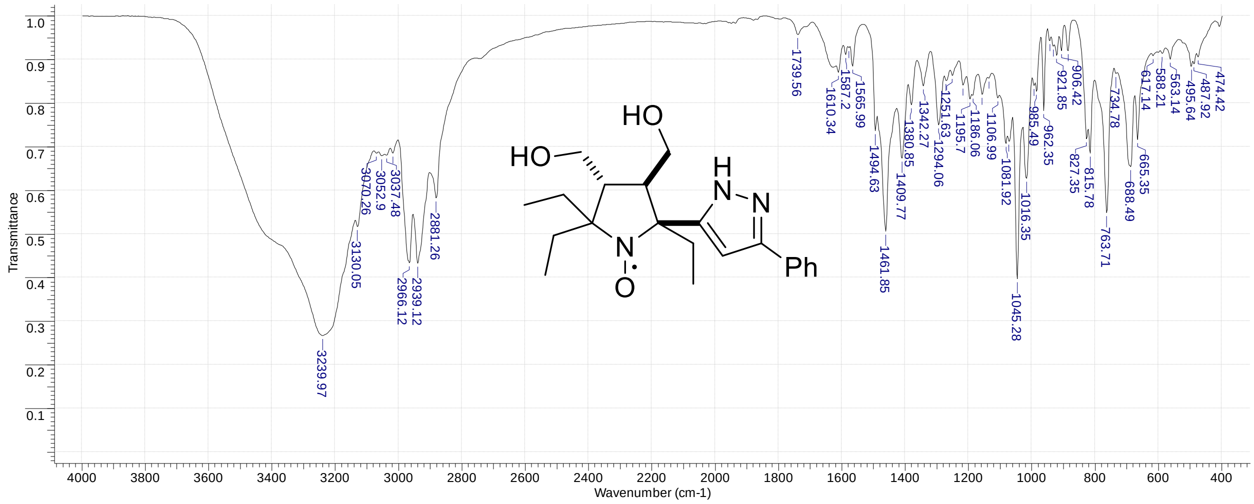

Figure S33. IR (neat) 3b,5,5-triethyl-6-(((methylsulfonyl)oxy)methyl)-2-phenyl-3b,4,5,6,6a,7-hexahydropyrrolo[2',3':3,4]pyrrolo[1,2-*b*]pyrazol-4-oxyl (9b)

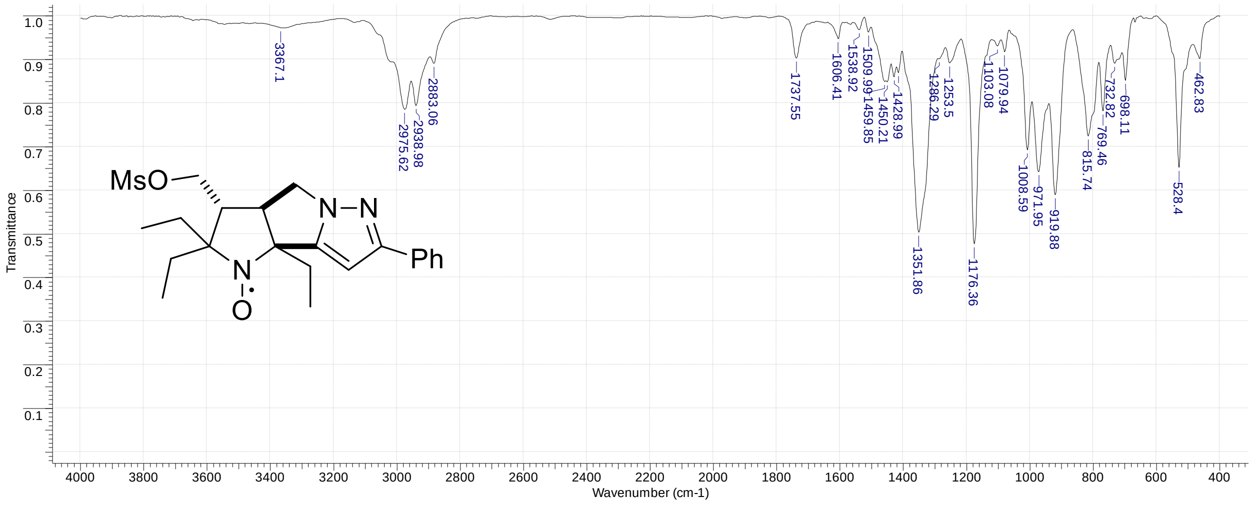

Figure S34. IR (KBr) 3b,5,5-triethyl-6-(hydroxymethyl)-2-phenyl-3b,4,5,6,6a,7-hexahydropyrrolo[2',3':3,4]pyrrolo[1,2-*b*]pyrazol-4-oxyl (9c)

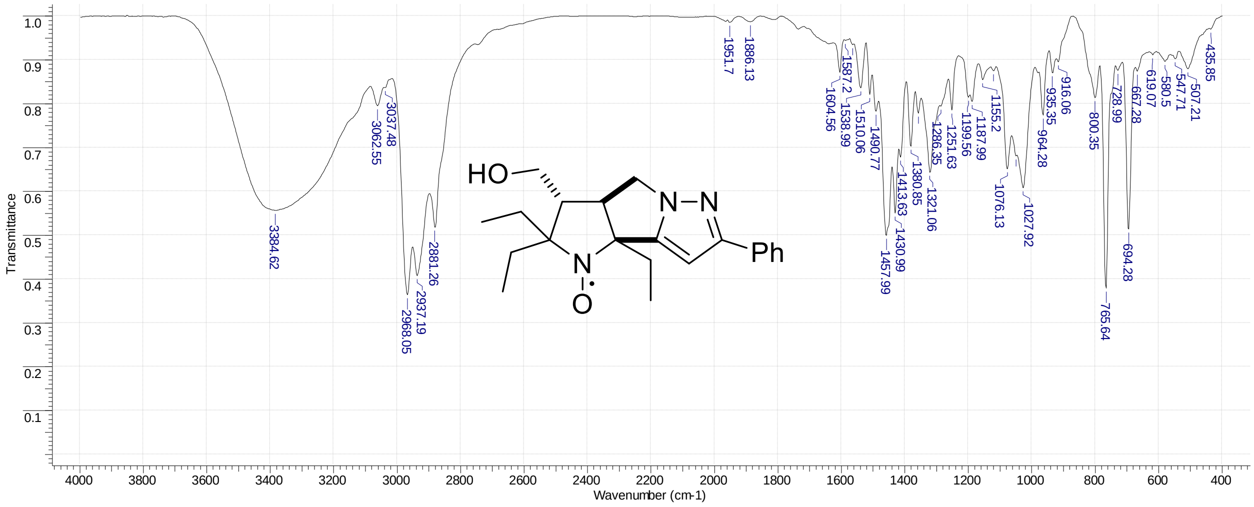

EPR measurements

CW EPR spectra were recorded in the X-band (≈9.87 GHz) using commercial Bruker Elexsys E 540 spectrometer (Bruker Corporation, Billerica, MA, USA). The measurements were performed in glass capillaries (100 μL) with the following spectrometer settings: modulation amplitude – 1 G; microwave power – 2 mW. Simulations of solution electron spin resonance lines were carried out in the EasySpin software (6.0.0), which is available at http://www.easyspin.org. EPR spectra were recorded for 0.4 mM buffer solutions of nitroxyl radicals.

The kinetics measurements were carried out using following general procedure. Fresh stock solutions of ascorbic acid (40, 80, 120 mM), glutathione (GSH) (8.0 mM), and a nitroxide (0.4 mM) in 10 mM phosphate buffer were prepared. The pH of the solutions was adjusted to 7.5 with NaOH (0.1 M solution). The stock solutions were carefully and quickly mixed using a stirrer Vortex V-1 Plus to achieve a concentration of 0.2 mM of the nitroxide, 10, 20 and 30 mM of ascorbate and 2 mM of glutathione. The mixture was taken into an EPR glass capillary. The sample was immediately placed into the resonator of the X-band CW EPR spectrometer and a series of spectra were recorded automatically one after another at equal intervals. Double integrals of the whole spectra were used to follow the nitroxide reduction. The data are plotted in Table S1 (see below). The analysis of the data was performed on the basis of previously published data on kinetics and mechanism of reduction of similar sterically shielded nitroxides in ascorbate–glutathione mixtures [S3]. The large excess of ascorbate allowed to process EPR signal decay rate as pseudo-first order reaction kinetics. The rate constants of the second-order reaction were determined by extracting the slope from the plot of the rate constant k_1 versus concentration of ascorbate. The measurement error was calculated based on the three kinetic points.

S3. Bobko, A. A.; Kirilyuk, I. A.; Grigor’ev, I. A.; Zweier, J. L.; Khramtsov, V. V. Reversible reduction of nitroxides to hydroxylamines: Roles for ascorbate and glutathione. *Free Radical Biol. Med.* **2006**, 42 (3), 404–412. https://doi.org/10.1016/j.freeradbiomed.2006.11.007.

Table S1. Reduction rate constants of nitroxide 2a–f, 4a–f, 9c

Nitroxide	$k_2 \cdot 10^3$, $M^{-1} s^{-1}$	Kinetic curves	k_1
**2a**	238.1 ± 10	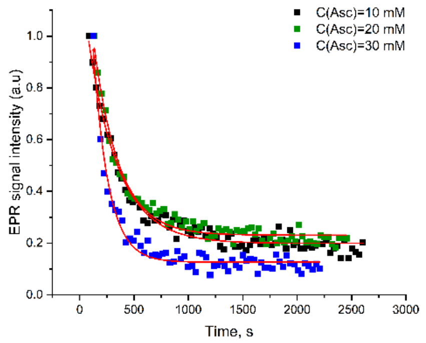	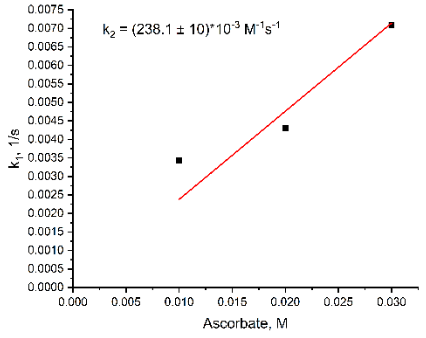
**2b**	185.6 ± 5	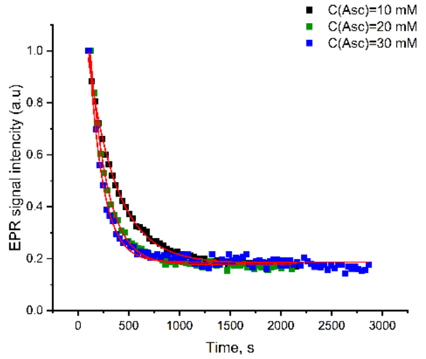	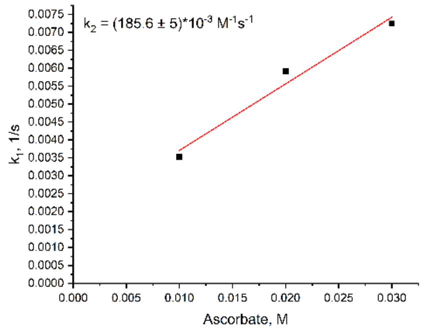
**2c**	135.7 ± 5	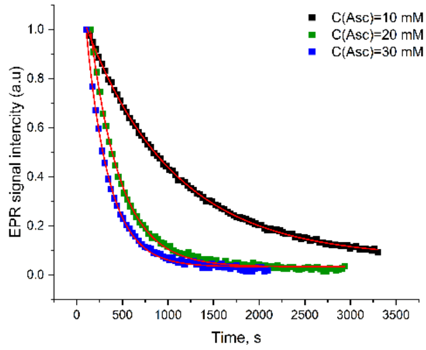	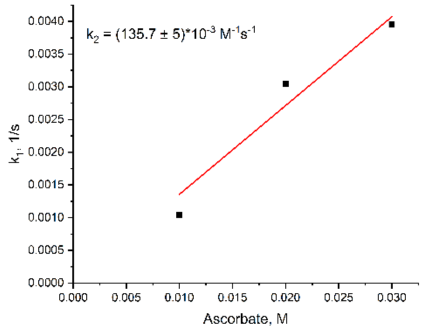
**2d**	250.0 ± 5	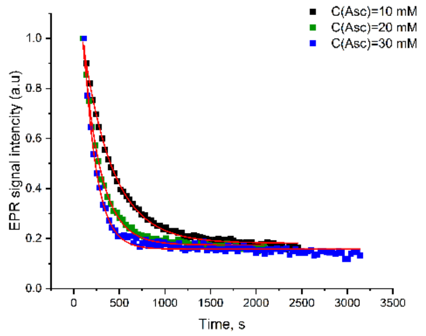	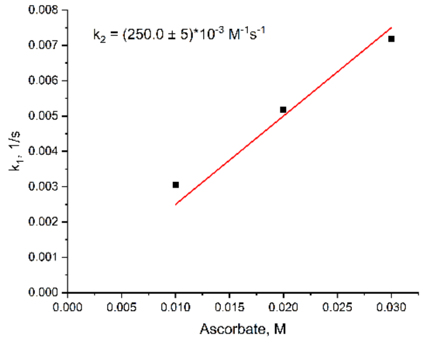

<table>
<tr><th>Nitroxide</th><th>$k_2 \cdot 10^3$, $M^{-1}s^{-1}$</th><th>Kinetic curves</th><th>k_1</th></tr>
<tr><td>2e</td><td>146.0 ± 6</td><td>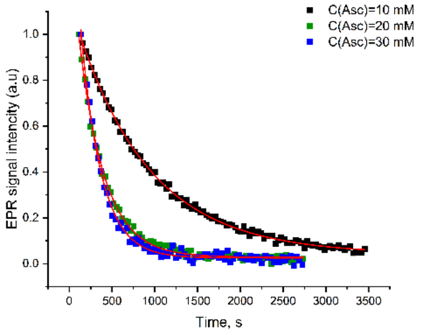
</td><td>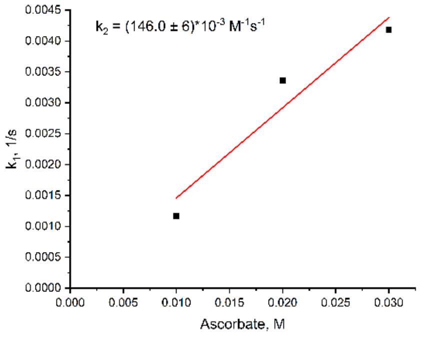
</td></tr>
<tr><td>2f</td><td>210.6 ± 5</td><td>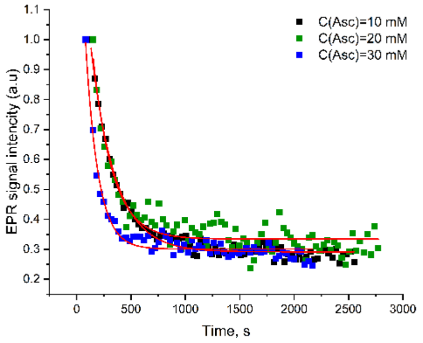
</td><td>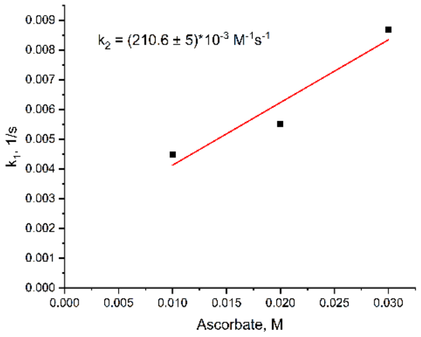
</td></tr>
<tr><td>4a</td><td>313.1 ± 2</td><td>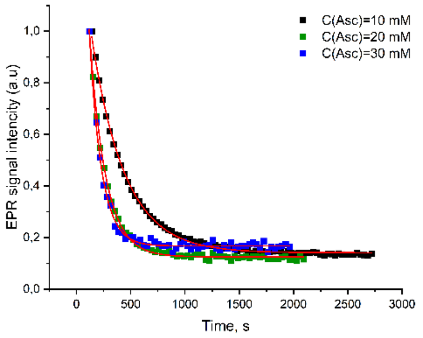
</td><td>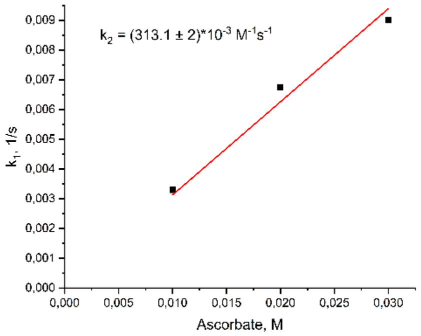
</td></tr>
<tr><td>4b</td><td>171.1 ± 6</td><td>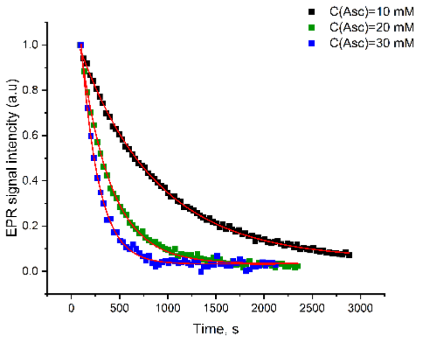
</td><td>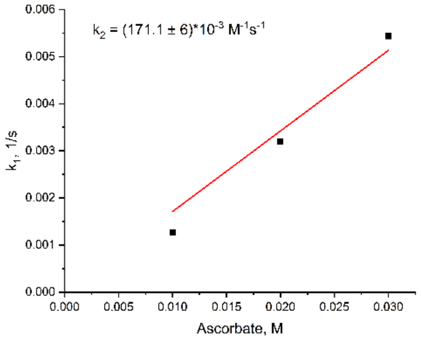
</td></tr>
</table>

<table>
<tr><th>Nitroxide</th><th>$k_2 \cdot 10^3$, $M^{-1}s^{-1}$</th><th>Kinetic curves</th><th>k_1</th></tr>
<tr><td>4c</td><td>439.2 ± 10</td><td>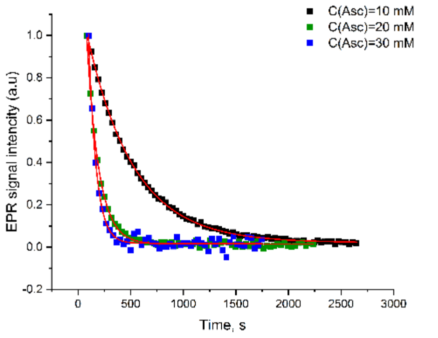
</td><td>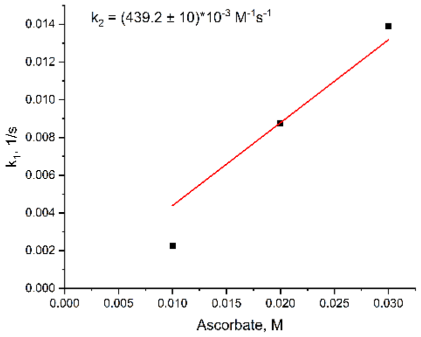
</td></tr>
<tr><td>4d</td><td>353.3 ± 5</td><td>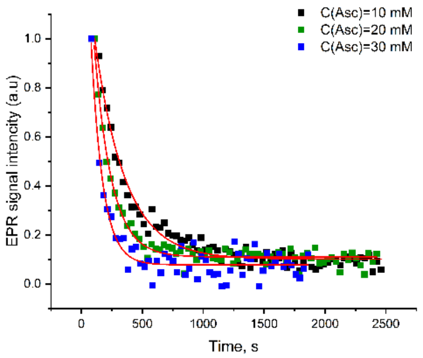
</td><td>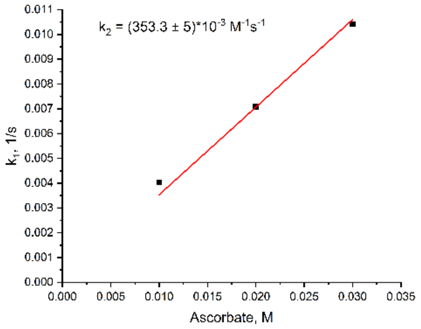
</td></tr>
<tr><td>4e</td><td>81.4 ± 1</td><td>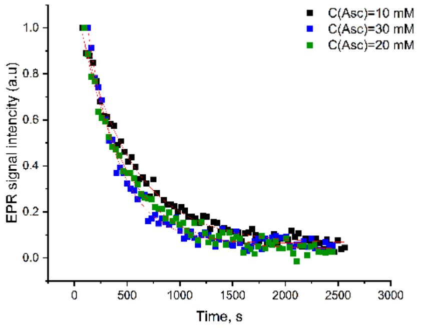
</td><td>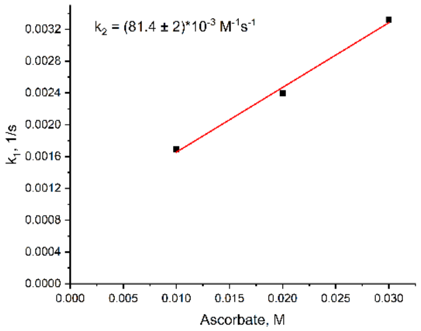
</td></tr>
<tr><td>4f</td><td>104.8 ± 5</td><td>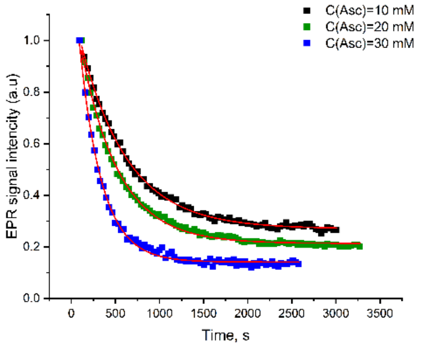
</td><td>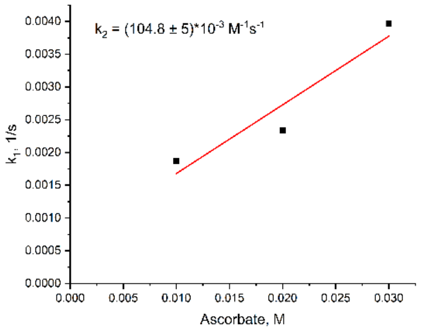
</td></tr>
</table>

Nitroxide	$k_2 \cdot 10^3$, $M^{-1} s^{-1}$	Kinetic curves	k_1
**9c**	85.2 ± 1	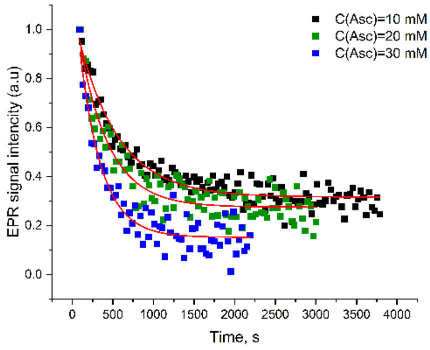	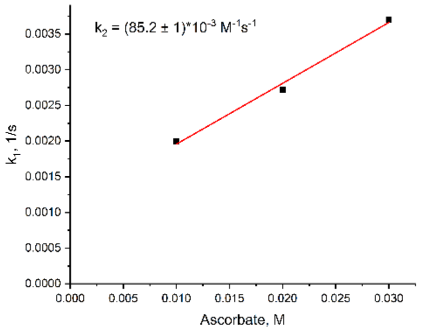

Table S2. EPR spectral parameters of nitroxides 2a–f, 4a–f, 9a–c

Nitroxide	a_N, mT (±0.005)	a_H, mT (±0.005)	Line width, mT (±0.001)	Spectrum
**2a**	1.53	0.23	0.006	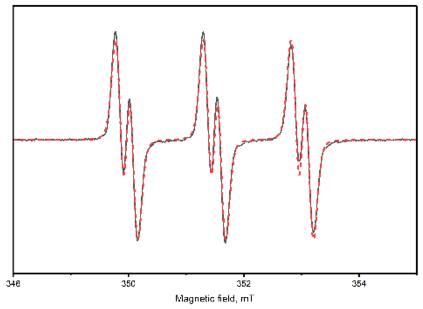
**2b**	1.53	0.21	0.006	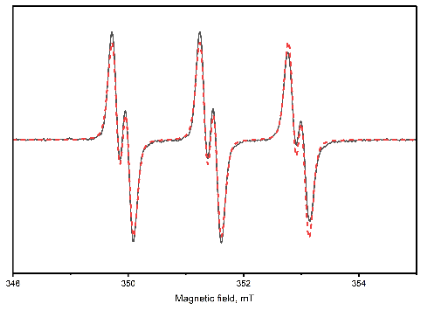
**2c**	1.54	0.21	0.006	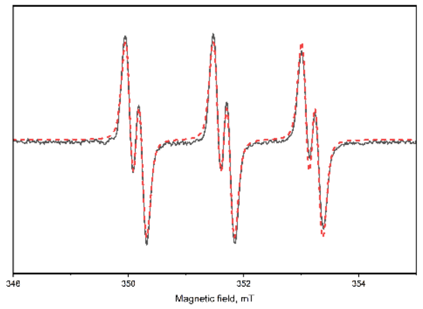

Nitroxide	a_N, mT (±0.005)	a_H, mT (±0.005)	Line width, mT (±0.001)	Spectrum
**2d**	1.53	0.21	0.006	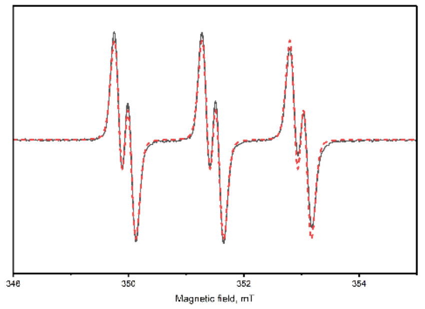
**2e**	1.53	0.21	0.006	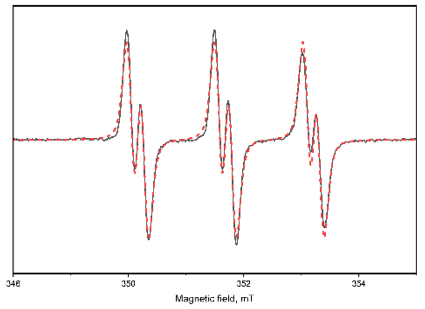
**2f**	1.53	0.23	0.006	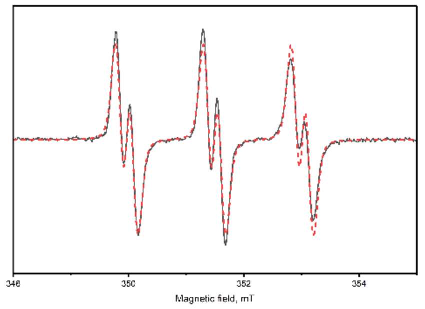
**4a**	1.47	0.26	0.007	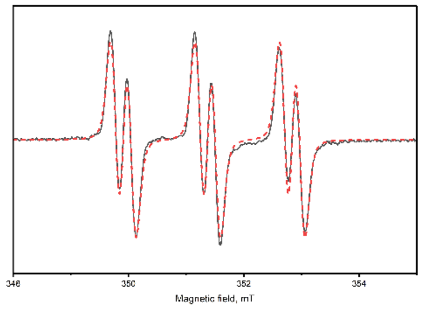
**4b**	1.45	0.22	0.007	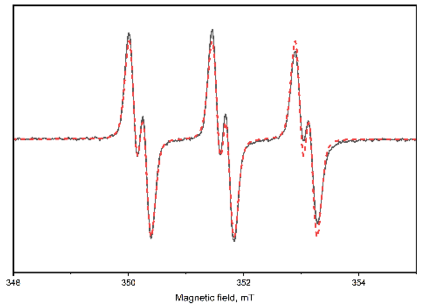

Nitroxide	a_N, mT (±0.005)	a_H, mT (±0.005)	Line width, mT (±0.001)	Spectrum
**4c**	1.45	0.27	0.007	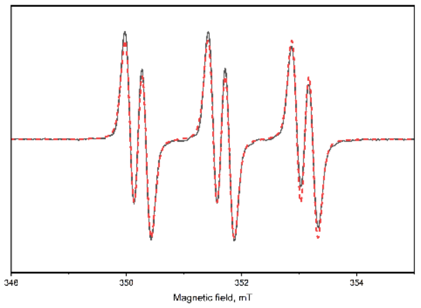
**4d**	1.45	0.26	0.007	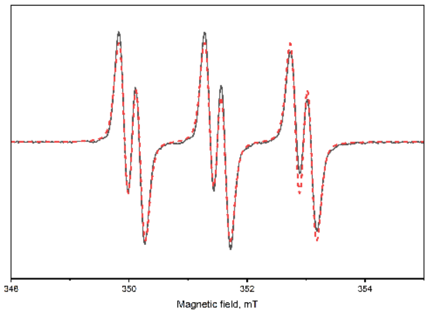
**4e**	1.43	0.26	0.007	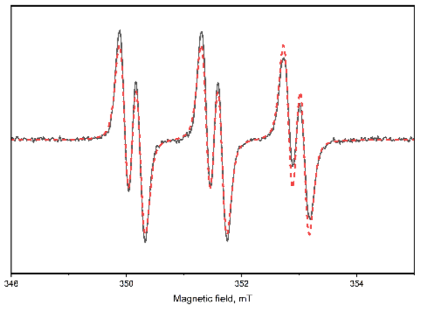
**4f**	1.46	0.31	0.006	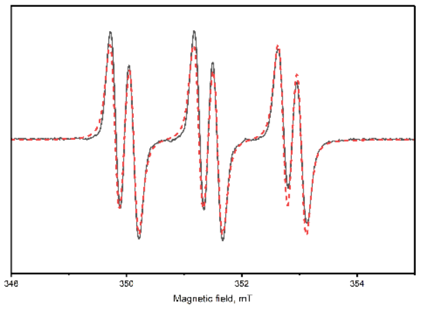
**9a**	1.50	0.28	0.007	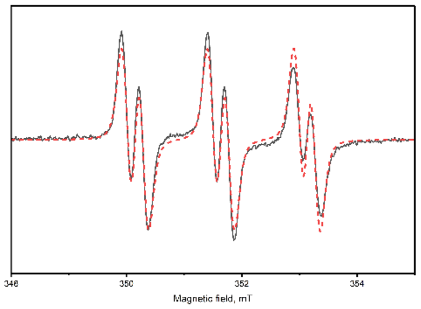

**Nitroxide**	***a*N, mT (±0.005)**	***a*H, mT (±0.005)**	**Line width, mT (±0.001)**	**Spectrum**
**9b**	1.47	0.26	0.007	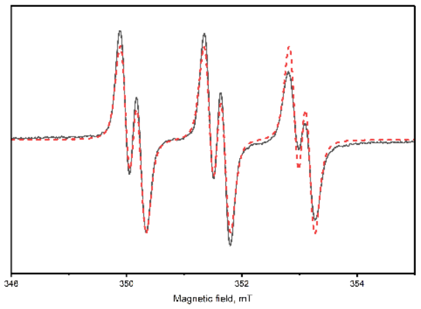
**9c**	1.49	0.25	0.007	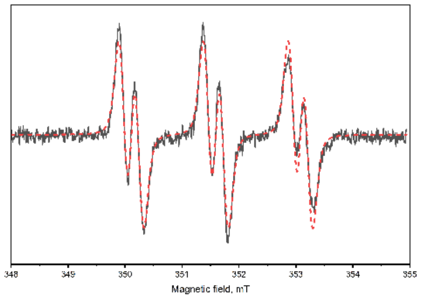

X-ray crystallographic analysis

The X-ray diffraction experiments were carried out on a Bruker KAPPA APEX II diffractometer (graphite-monochromated Mo Kα radiation). Reflection intensities were corrected for absorption by *SADABS2016*/2 program [S4]. The structure was solved by direct methods using the *SHELXT*2014/5 [S5] and refined by anisotropic (isotropic for all H atoms) full-matrix least-squares method against F^2 of all reflections by *SHELXL2018*/3[S6]. The positions of the hydrogen atoms were calculated geometrically and refined in a riding model except for **2d**, **8** and **9**. Asymmetric unit of **2d** includes four independent molecules and for one of them hydroxy hydrogens were localized from d-map and refined independently with O–H bond lengths restriction to 0.82 Å. The same procedure was done for hydroxyls of **6** and **9a** without any restriction of bond lengths. The details of experiment and structure refinement are given in Table S3.

Crystallographic data for **2d**, **4a–c**, **8**, **9a** have been deposited at the Crystallographic Data Centre as supplementary publication no. CCDC 2512649-2512654. Copy of the data can be obtained, free of charge, on application to CCDC, 12 Union Road, Cambridge CB21EZ, UK (fax: +44 122 3336033 or e-mail: deposit@ccdc.cam.ac.uk; internet: www.ccdc.cam.ac.uk).

Table S3. Experimental and structure refinement details for 2d, 4a–c, 8, 9a

	**2d**	**4a**	**4b**	**4c**	**8**	**9a**
Chemical formula	$C_{17}H_{32}NO_3Si$	$C_{14}H_{22}N_7O$	$C_{20}H_{26}N_7O$	$C_{15}H_{23}N_{10}O$	$C_{15}H_{24}NO_4$	$C_{21}H_{30}N_3O_3$
M_r	326.52	304.38	380.48	359.43	282.35	372.48
Crystal system, space group	Triclinic, $P\bar{1}$	Monoclinic, $P2_1$	Monoclinic, $P2_1/n$	Monoclinic, $P2_1/c$	Monoclinic, $P2_1/n$	Monoclinic, $P2_1/n$
Temperature (K)	296	296	296	296	296	200
a, *b*, *c* (Å)	12.4707(5) 17.5991(8) 19.5933(9)	7.4655(5) 11.8859(6) 9.1833(5)	8.403(3) 8.881(3) 27.00(1)	12.2895(7), 9.5375(4), 15.8665(7)	8.0418(7), 12.055(1), 15.989(2)	14.501(3), 10.311(2), 14.717(3)
α, β, γ (°)	82.553(2) 79.474(2) 71.963(2)	90, 95.219(2), 90	90, 93.113(10), 90	90, 98.802(2), 90	90, 102.711(4), 90	90, 115.511(6), 90
V (Å^3)	4007.8 (3)	811.49 (8)	2012.0 (13)	1837.83 (15)	1512.0(3)	1985.9(7)
Z	8	2	4	4	4	4

F(000)	1432	326	812	764	612	804
D_x (Mg m^{-3})	1.082	1.246	1.256	1.299	1.240	1.246
μ(mm^{-1})	0.13	0.09	0.08	0.09	0.09	0.08
Crystal size (mm)	0.80 × 0.40 × 0.20	0.72 × 0.41 × 0.31	0.41 × 0.24 × 0.07	0.68 × 0.58 × 0.25	0.29 × 0.13 × 0.04	0.40 × 0.10 × 0.06
T_{min}, T_{max}	0.707, 0.745	0.877, 0.928	0.909, 0.959	0.897, 0.937	0.883, 0.958	0.901, 0.971
No. of measured, independent and observed [$I > 2\sigma(I)$] reflections	56190, 15904, 10265	14376, 4103, 3584	43855, 4848, 3551	30287, 4221, 3182	9999, 2661, 1428	23165, 3507, 2299
R_{int}	0.035	0.045	0.069	0.060	0.079	0.091
θ range (°)	1.6 - 26.1	2.7 - 28.5	2.8 - 28.0	2.9 - 27.5	3.1 - 25.1	3.3 - 25.1
Range of *h*, *k*, *l*	*h* = -15→15 *k* = -21→21 *l* = -24→24	*h* = -9→10 *k* = -15→15 *l* = -12→12	*h* = -11→11 *k* = -11→11 *l* = -35→35	*h* = -15→15 *k* = -12→12 *l* = -20→20	*h* = -8→9 *k* = -14→13 *l* = -18→19	*h* = -17→17 *k* = -12→12 *l* = -17→17
$R[F^2 > 2\sigma(F^2)]$, $wR(F^2)$	0.064, 0.215	0.049, 0.140	0.049, 0.139	0.051, 0.165	0.077, 0.230	0.045, 0.111
Goof	1.00	1.05	1.02	1.02	1.00	1.00
No. of reflections	15904	4103	4848	4221	2661	3507
No. of parameters	840	202	253	238	188	259
No. of restraints	1	1	0	0	0	0
ρ_{max}, ρ_{min} (e Å^{-3})	1.57, -0.49	0.29, -0.20	0.25, -0.18	0.35, -0.28	0.46, -0.18	0.18, -0.24
Flack parameter	–	0.0 (6)	–	–	–	–
CCDC number	2512649	2512650	2512651	2512652	2512653	2512654

S4. SADABS, v. 2008-1, Bruker AXS, Madison, WI, USA

S5. Sheldrick, G. M. SHELXT– Integrated Space-Group and Crystal-Structure Determination. *Acta Crystallogr., Sect. A*, **2015**, 71 (1), 3–8. https://doi.org/10.1107/s2053273314026370.

S6. Sheldrick, G. M. Crystal Structure Refinement WithSHELXL. *Acta Crystallogr. Sect. C.* **2015**, 71 (1), 3–8. https://doi.org/10.1107/s2053229614024218.
